# From haemadin to haemanorm: Synthesis and characterization of full‐length haemadin from the leech *Haemadipsa sylvestris* and of a novel bivalent, highly potent thrombin inhibitor (haemanorm)

**DOI:** 10.1002/pro.4825

**Published:** 2023-12-01

**Authors:** Laura Acquasaliente, Andrea Pierangelini, Anna Pagotto, Nicola Pozzi, Vincenzo De Filippis

**Affiliations:** ^1^ Laboratory of Protein Chemistry & Molecular Hematology, Department of Pharmaceutical and Pharmacological Sciences, School of Medicine University of Padova Padua Italy; ^2^ Department of Biochemistry and Molecular Biology, Edward A. Doisy Research Center Saint Louis University St. Louis Missouri USA

**Keywords:** coagulation, haemadin, HDX‐MS, hirudin, molecular recognition, natural anticoagulants, noncoded amino acids, peptide synthesis, protease inhibitors, thrombin

## Abstract

Hirudin from *Hirudo medicinalis* is a bivalent α‐Thrombin (αT) inhibitor, targeting the enzyme active site and exosite‐I, and is currently used in anticoagulant therapy along with its simplified analogue hirulog. Haemadin, a small protein (57 amino acids) isolated from the land‐living leech *Haemadipsa sylvestris*, selectively inhibits αT with a potency identical to that of recombinant hirudin (*K*_I_ = 0.2 pM), with which it shares a common disulfide topology and overall fold. At variance with hirudin, haemadin targets exosite‐II and therefore (besides the free protease) it also blocks thrombomodulin‐bound αT without inhibiting the active intermediate meizothrombin, thus offering potential advantages over hirudin. Here, we produced in reasonably high yields and pharmaceutical purity (>98%) wild‐type haemadin and the oxidation resistant Met5 → nor‐Leucine analogue, both inhibiting αT with a *K*_I_ of 0.2 pM. Thereafter, we used site‐directed mutagenesis, spectroscopic, ligand‐displacement, and Hydrogen/Deuterium Exchange‐Mass Spectrometry techniques to map the αT regions relevant for the interaction with full‐length haemadin and with the synthetic N‐ and C‐terminal peptides Haem(1–10) and Haem(45–57). Haem(1–10) competitively binds to/inhibits αT active site (*K*_I_ = 1.9 μM) and its potency was enhanced by 10‐fold after Phe3 → β‐Naphthylalanine exchange. Conversely to full‐length haemadin, haem(45–57) displays intrinsic affinity for exosite‐I (*K*_D_ = 1.6 μM). Hence, we synthesized a peptide in which the sequences 1–9 and 45–57 were joined together through a 3‐Glycine spacer to yield haemanorm, a highly potent (*K*_I_ = 0.8 nM) inhibitor targeting αT active site and exosite‐I. Haemanorm can be regarded as a novel class of hirulog‐like αT inhibitors with potential pharmacological applications.

## 1 INTRODUCTION

**FIGURE 1:**
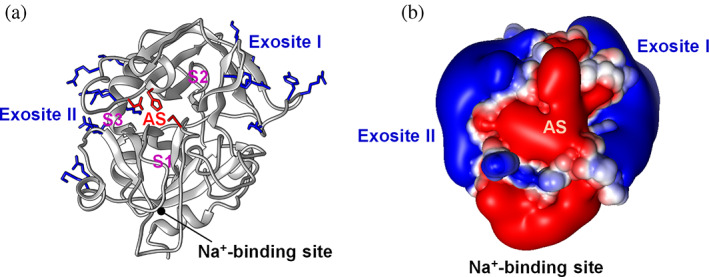
Ribbon drawing (a) and electrostatic potential surface (b) representation of αT structure. (a) Structure of αT, as deduced from the crystallographic structure of the enzyme inhibited with PPACK (1 ppb), after removal of the inhibitor. Basic amino acids in the positively charged exosite I (K36, R67, R73, Y76, R77a, K81) and exosite II (R93, R101, R126, R165, K169, R233, K235, K236, K240) are colored blue, while the catalytic amino acids in the active site (H57, D102, and S195) are in red. The position of the substrate specificity sites S1, S2 and S3 is tentatively indicated, along with the Na^+^‐binding site, which is contributed by the 180‐loop and 220‐loop. The primary specificity S1 site contains D189 at the bottom and determines enzyme specificity for Arg‐containing substrates at P1 site; the S2 site is shaped by the 60‐loop, stereochemically constraining the size of the side chain at P2 position; the S3 site is mainly contributed by the apolar amino acids L99, I174 and W215. For macromolecular substrates and inhibitors targeting, αT also exploits exosite I and exosite II. Exosite I is shaped by the 30‐loop and 70‐loop while exosite II is contributed by the outer surface of 90‐loop and by the C‐terminal helices of αT B‐chain. (b) Surface electrostatic potential of αT. The surface is colored according to the electrostatic potential (blue, positive; red, negative) and expressed as kJ/(mol·q). The active‐site (AS) and surrounding region are negatively charged, whereas the exosites are strongly positive. Calculations were performed using the APBS program, run on the coordinates of αT‐PPACK, after removal of inhibitor atoms.

Thrombotic diseases may be idiopathic or appear as the expression of thrombotic complications, occurring with variable incidence and severity in different (apparently unrelated) diseases, such as for instance type‐2 diabetes (Lancellotti et al., 2010; Pozzi et al., 2012), chronic kidney disease (De Filippis et al., 2012), inflammatory bowel disease (Pontarollo et al., 2017), cancer (Patmore et al., 2020), rheumatoid arthritis (Sokolov et al., 2015), autoimmune diseases (Acquasaliente et al., 2016; Pozzi et al., 2013), amyloidosis (Peterle et al., 2020; Acquasaliente & De Filippis, 2022), and bacterial (Levi et al., 1999; Pontarollo et al., 2017) and viral (Iba et al., 2020) infections. Most frequently, they are characterized by aberrant generation of active α‐thrombin (αT) (Di Cera, 2008), a serine protease (36 kDa) that plays an important role at the interface between coagulation, inflammation, and cell growth (Esmon et al., 1999), and exerts procoagulant and anticoagulant functions in hemostasis (Di Cera, 2007). The procoagulant role entails conversion of soluble fibrinogen into insoluble fibrin and proteolytic activation of platelets via protease‐activated receptor (PAR) cleavage, whereas the anticoagulant role of αT involves the thrombomodulin (TM)‐assisted activation of Protein C, which in turn proteolytically degrades the procoagulant co‐factors Va and VIIIa. The equilibrium between the pro‐coagulant and anti‐coagulant functions of αT is mainly regulated by effector proteins interaction and, to a minor extent, by sodium ion binding at a specific protease site (Figure 1). Na^+^ binding triggers the conformational transition of the enzyme from an anticoagulant “slow” form to a procoagulant “fast” form (Dang et al., 1995; Lechtenberg et al., 2010; Pelc et al., 2022). The Na^+^‐bound (fast) form displays procoagulant properties, since it cleaves more specifically fibrinogen and PAR‐1, whereas the Na^+^‐free (slow) form is anticoagulant as it retains normal proteolytic activity toward Protein C, but is unable to promote physiologically acceptable hydrolysis of the procoagulant substrates (Dang et al., 1995; De Filippis et al., 2005).

αT structure is that typical of the chymotrypsin superfamily fold of serine proteases, with two six‐stranded β‐barrels that pack together asymmetrically to accommodate at their interface the catalytic amino acids triad (Figure 1) (Bode et al., 1992). For macromolecular substrates and inhibitors targeting, besides the active site and the substrate specificity sites, αT exploits two positively charged exosites, that is, exosite I and exosite II. Exosite I binds both pro‐coagulant (fibrinogen and PAR1) substrates and anti‐coagulant (TM) physiological co‐factors, while exosite II interacts with negatively charged ligands/inhibitors like heparin and heparan sulphate, the prothrombin F2 fragment, the fibrinogen elongated γ‐chain, and the C‐terminal tail of platelet receptor glycoprotein Ibα. Furthermore, both exosites variably contribute in the co‐factor V and VIII binding/activation. Exosite II is flatter and more electropositive than exosite I, and ligand binding is mainly driven by less specific electrostatic charge–charge complementarity, while exosite I requires more specific interactions for molecular recognition (Bock et al., 2007; Huntington, 2005).

Considering its pivotal role in hemostasis, αT has become a primary target for the development of novel and safer anticoagulants of both natural (Corral‐Rodríguez et al., 2010; Huntington, 2014; Lombardi et al., 1999) and synthetic (Lee & Ansell, 2011) origin. The main source of natural anticoagulants is represented by blood‐feeding parasites (hematophagous) that, to overcome host response systems (i.e., activation of blood coagulation), produce highly potent small protein proteinase inhibitors, most often selectively targeting αT (Corral‐Rodríguez et al., 2010; De Filippis, Acquasaliente, et al., 2018; Huntington, 2014). Natural hirudin HV1 variant, extracted from the aquatic leech *Hirudo medicinalis*, along with its recombinant derivatives lepirudin (Refludan®) and desirudin (Revasc®/Iprivask®), was the first naturally occurring αT inhibitor to be approved by the European Agency for the Evaluation of Medicinal Products and the US Food and Drug Administration for the treatment of heparin induced thrombocytopenia (HIT) and associated thrombotic disease. Desirudin is currently used to prevent proximal deep vein thrombosis in patients undergoing hip or knee arthroplasty (Graetz et al., 2011), whereas bivalirudin (Angiomax®) (i.e., a synthetic bivalent analogue of hirudin) is used in the treatment of venous thromboembolism and HIT in adults, and in the systemic anticoagulation in pediatric patients (Capranzano & Dangas, 2012).

The relevance of pharmacological applications of hirudin and its analogues has stimulated intensive research on hematophagous organisms, allowing identification of novel αT protein inhibitors, including the isolation of haemadin from the Indian leech *Haemadipsa sylvestris* (Strube et al., 1993). Haemadin is a 57‐amino acid polypeptide that selectively inhibits αT with a potency (*K*_I_ ~ 0.2 pM) identical to that of recombinant hirudin, with which it shares a common disulfide topology and an overall fold. Haemadin and hirudin, in fact, contain a compact N‐terminal domain, stabilized by three SS bonds, and a highly negative and flexible C‐terminal tail. Comparative analysis of αT complexed to hirudin (Rydel et al., 1991) or haemadin (Richardson et al., 2000) shows that both inhibitors use the N‐terminal domain to block the protease active site, whereas their C‐terminal tail binds to different exosites. While crystallographic analysis safely indicates that hirudin interacts with exosite I, the available crystal structure of the haemadin‐αT complex, solved at low resolution (3.1 Å), shows the presence of three molecules in the asymmetric unit cell, each with a 1:1 haemadin:αT stoichiometry (Richardson et al., 2000). In the first and third molecule, the N‐terminal region of haemadin penetrates into αT active site, whereas the negative C‐terminal region binds to exosite II. In the second molecule, while the interaction of the N‐terminal region with the active site is confirmed, the C‐terminal tail is found to interact with the exosite I of the neighboring αT molecule.

In this study, we report the total chemical synthesis and the conformational, stability, and functional characterization of haemadin. Using site‐directed mutagenesis, spectroscopic and hydrogen/deuterium exchange mass spectrometry (HDX‐MS) techniques, we mapped αT regions relevant for the interaction with full‐length haemadin and with synthetic peptides corresponding to the N‐terminal (amino acid residues 1–10) and C‐terminal (amino acid residues 45–57) regions of haemadin. Finally, the results of molecular mapping and functional dissection of haemadin structure were used to synthesize a bivalent and highly potent (*K*_I_ = 0.8 nM) αT peptide inhibitor, hereafter denoted as haemanorm.

## 2 RESULTS

### 2.1 Synthesis, disulfide oxidative refolding and chemical characterization of wild‐type haemadin and its Met5 → nLeu analogue

**FIGURE 2:**
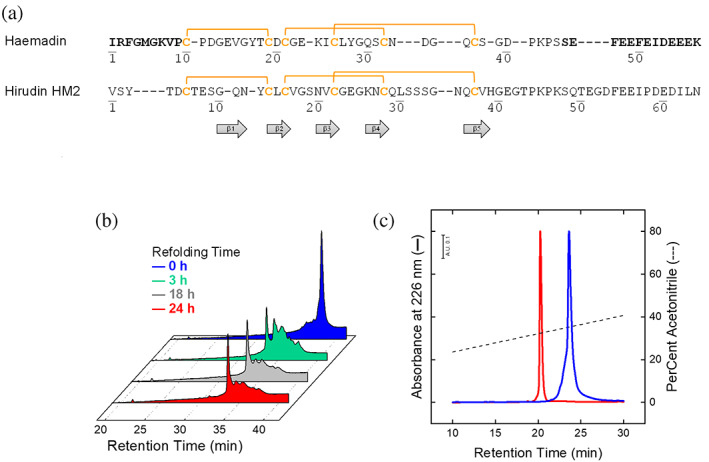
Chemical synthesis, purification, and disulfide oxidative renaturation of haemadin 1–57. (a) Amino acid sequence of haemadin from *Haemadipsa sylvestris* and hirudin HM2 from *Hirudinaria manillensis*. The N‐Terminal (amino acids 1–10) and the C‐terminal region (amino acids 45–57) of haemadin are represented in bold, cysteine residues are in yellow, and disulfide bonds are indicated by plain lines. (b) Time course RP‐HPLC analysis of the oxidative disulfide renaturation of haemadin. Fully reduced, HPLC‐purified peptide haemadin (0.4 mg/mL) was allowed to fold at 25°C in 0.1 M Tris–HCl buffer pH 8.3 in the presence of 250 μM β‐ME. At time intervals (0, 3, 18 and 24 h) aliquots (100 μg) of the refolding mixture were acid quenched and analyzed by RP‐HPLC. (c) RP‐HPLC analysis of purified, folded haemadin, containing oxidized disulfide bonds (N‐Haem). An aliquot (10 μg) of N‐Haem (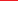) was injected into a Zorbax C18 analytical column (4.6 mm × 150 mm), eluted with a linear acetonitrile gradient (‐ ‐ ‐) in 0.1% (v/v) aqueous TFA, from 15% to 45% in 30 min, at a flow rate of 0.8 mL/min. For comparison, the RP‐HPLC analysis of fully disulfide reduced haemadin is also reported (R‐Haem; 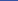). The peptide material eluted with N‐Haem or R‐Haem peaks was collected and analyzed by HR‐MS, yielding mass values in agreement with the amino acid composition of the reduced and oxidized species within 10 ppm mass accuracy (see Table S1).

The chemical synthesis of full‐length wild‐type haemadin 1–57 (Figure 2a) was carried out using the standard stepwise Fmoc/tBu chemistry in solid‐phase peptide synthesis (SPPS), as detailed in Section 5 (see also Figure S1). The crude peptide, with Cys‐residues in the reduced form (R‐Haem) was purified by semi‐preparative RP‐HPLC, lyophilized and subjected to oxidative disulfide folding reaction. Although different additives were tested to improve renaturation yields, the best conditions for R‐Haem renaturation were 24 h‐reaction at 25° in 0.1 M Tris–HCl buffer, pH 8.3, containing 250 μM β‐mercaptoethanol (β‐ME), and at a protein concentration of 0.5 mg/mL. RP‐HPLC analysis of the time‐course disulfide oxidative reaction (Figure 2b) shows the progressive accumulation of a species eluting at shorter r.t., compatible with the less apolar surface that the folded species (N‐Heam) is expected to expose to the RP‐column, and with a molecular mass (6252.25 ± 0.3 a.m.u.) six units lower than that of R‐Haem (6257.74 ± 0.02 a.m.u.), consistent with the formation of the three disulfide bonds present in the native haemadin (Figure 2a). Additionally, N‐Haem did not react with the free Cys‐specific 5,5′‐dithiobis (2‐nitrobenzoic acid) reagent (not shown), further confirming complete disulfide bridging in N‐Haem. N‐Haem was then purified by semipreparative RP‐HPLC (Figure 2c) and subjected to proteolysis with several different proteases to chemically assign disulfide bond pairings. However, in the case of N‐Haem, the highly cross‐linked nature of the N‐terminal domain made this strategy of limited success, yielding only nicked (still crosslinked) species (not shown) that practically impaired S–S bonds assignment. Notably, during the disulfide folding reaction of wild‐type haemadin (Figure 2b), the species eluting at longer r.t. have a molecular mass identical to that of N‐haem, but only limited (if any) inhibitory effect on αT amidolytic activity, which was tested on the chromogenic substrate S‐2238 (not shown), thus suggesting that these species correspond to misfolded (disulfide‐scrambled) forms of haemadin. The addition of relatively high β‐ME concentrations was found beneficial for folding, making non‐native (less stable) S–S bonds to be more easily reduced, compared to the (more stable) native S–S bonds, thus allowing the polypeptide chain to explore additional possibilities to fold into the correct native‐like structure (Chatrenet & Chang, 1993; De Filippis et al., 1995; De Filippis et al., 1998). The overall final yield of pure haemadin (1–57) (comprising stepwise synthesis, polypeptide resin cleavage, purification of R‐Haem, disulfide‐oxidative renaturation and final purification of N‐Haem) was found >10%, reasonably high if compared with the much lower yields (0.1%–4%) of other disulfide cross‐linked peptides of similar size (Jin et al., 2019; Tombling et al., 2020).

Taking advantage of the versatility of SPPS and the analytical methods previously established for the chemical characterization of the wild‐type inhibitor, here we produced an analogue of full‐length haemadin (Met5*n*Leu) in which the methionine at position 5 was replaced with the non‐natural amino acid *nor‐*leucine (*n*Leu). Whereas Met can undergo oxidative reactions to sulfoxide or sulfone in proteins (Brot & Weissbach, 1983), *n*Leu is oxidation‐resistant while sharing with Met similar size, hydrophobicity and conformational properties (Fauchère et al., 1988). Both wild‐type and Met5*n*Leu haemadins were purified in sufficient amounts for further conformational, stability, and functional characterization.

### 2.2 Conformational and stability properties of wild‐type haemadin and comparison with hirudin

**FIGURE 3:**
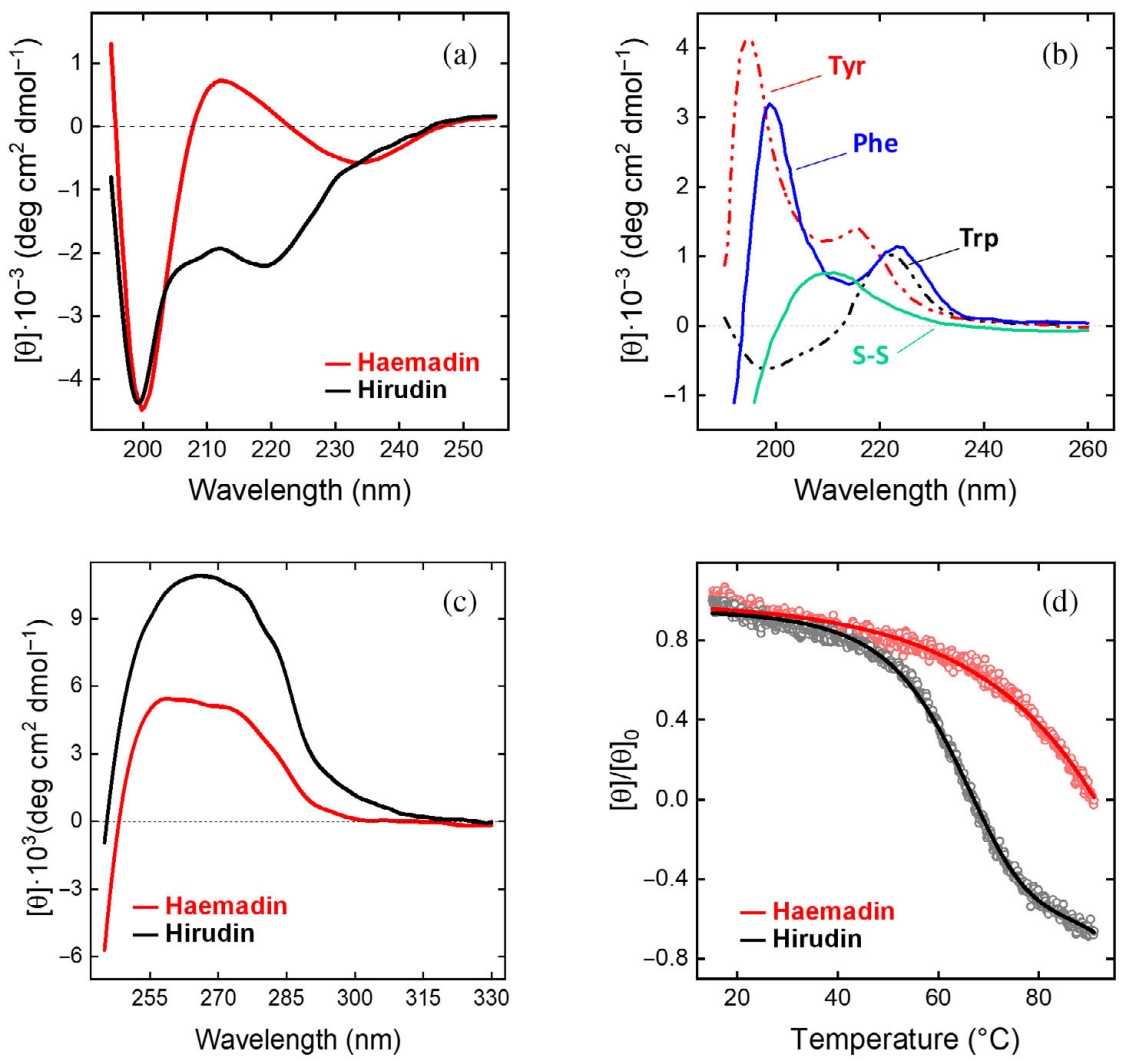
Comparative analysis of the conformational and stability properties of haemadin and hirudin HM2. Far‐UV (a) and near‐UV CD (c) spectra of haemadin (red curves) and hirudin (black curves). Spectra were recorded at 25 ± 0.1°C, in PBS, at a protein concentration of 0.47 and 1 mg/mL in the far‐ and near‐UV region, respectively, and the resulting spectra were corrected for the corresponding base lines. (b) Far‐UV CD spectra of model compound solutions of cystine and N^α^‐acetyl‐amide derivatives of Tyr, Phe and Trp, as indicated. CD signal for model compound solutions are expressed as molar ellipticity. (d) Thermal denaturation of haemadin and hirudin HM2. Measurements were carried out in PBS at a protein concentration of 0.69 mg/mL, by recording the change in the CD signal at 233 nm for haemadin and 220 nm for hirudin. The melting temperature (*T*_m_) was estimated as the inflection point of the denaturation curve and found as 63 ± 1°C for hirudin and of 85 ± 2°C for haemadin.

The circular dichroism (CD) spectra of synthetic haemadin in the far‐ and near‐UV region were compared with those of the structurally homologous hirudin, with which haemadin shares a common fold, but only limited (32.9%) amino acid sequence similarity (Figure 2a). The far‐UV CD spectra of both haemadin and hirudin (Figure 3a) show an intense negative band at 200 nm, assigned to the disordered/flexible C‐terminal tail present in the two inhibitors. Whereas the spectrum of hirudin is characterized by a minimum at 220 nm, consistent with the protein β‐sheet content (De Filippis et al., 1998; Polverino de Laureto et al., 1999), haemadin displays an unusual spectrum with a positive band at 212 nm and a negative signal centered at 230 nm. Likely, these differences reflect the different non‐conformational contribution in the far‐UV region of the aromatic amino acids in hirudin (i.e., 2 Tyr, 1 Phe) and haemadin (i.e., 2 Tyr, 3 Phe), which in proteins with low secondary structure content can overwhelm the conformational contribution of the peptide bonds absorption, as shown from the spectra of aromatic amino acids model compounds (Figure 3b).

Near‐UV CD spectra of both haemadin and hirudin are dominated by the contribution of disulfide bonds, appearing as a broad positive band in the 250–290 nm region (Figure 3c). Notably, right‐ or left‐handed S–S bonds give positive and negative CD signals, respectively (De Filippis et al., 2002). Hence, the positive signal in the spectrum of hirudin is consistent with the right‐handed chirality that all three S–S bonds assume in the hirudin structure (Folkers et al., 1989; Rydel et al., 1991). Likewise, the lower intensity of the same band in the spectrum of haemadin reflects the fact that only two S–S bonds (i.e., Cys21‐Cys32; Cys26‐Cys37) have a right‐handed chirality, whereas Cys10‐Cys19 bond is in a left‐handed conformation (Richardson et al., 2000).

The thermal denaturation of haemadin was monitored by recording the decrease in the CD signal in the far‐UV region as a function of temperature, in the 15–91°C range, and compared to that of hirudin (Figure 3d). In both cases, denaturation is fully reversible, as judged from the recovery of the ellipticity value upon cooling at the starting temperature, but the lack of a clear post‐transition region in the melting curves limits the possibility of performing a rigorous thermodynamic treatment, and only a rough estimate of the melting temperature (*T*_m_) could be extracted from the inflection point of the denaturation profile. Notably, the *T*_m_ value of haemadin was estimated as 85 ± 2°C, approximately 22°C higher than that of hirudin (*T*_m_ = 63 ± 1°C).

### 2.3 Inhibition of αT amidolytic activity by wild‐type haemadin and its Met5nLeu analogue

The ability of synthetic haemadins to inhibit αT amidolytic activity was tested at 20°C by measuring the release of *p*‐nitroaniline (*p*NA) from the chromogenic substrate (D)‐Phe‐Pip‐Arg‐pNA (S2238), under conditions of tight binding or slow binding inhibition (Richardson et al., 2002) and in the presence of 0.2 M NaCl or choline chloride (ChCl), stabilizing the fast or slow form of thrombin (Dang et al., 1995). In the case of tight binding, the substrate was added to a solution of αT pre‐incubated for 30 min with increasing haemadin concentrations (Figure S2_A). Analysis of steady state velocity data as a function of [haemadin] allowed us to calculate an inhibition constant (*K*_I_) of 0.23 ± 0.01 pM and 1.50 ± 0.05 pM for the fast and slow form, respectively, where *K*_I_(fast) is identical to that previously determined for natural (Richardson et al., 2002; Strube et al., 1993) and recombinant (Kostromina et al., 2021; Richardson et al., 2002) haemadin, measured at 25°C and 0.2 M NaCl. Notably, the values of *K*_I_(fast) and *K*_I_(slow) of the Met5nLeu analogue were only 2‐fold higher than those measured for the wild‐type inhibitor, suggesting that the conservative Met → nLeu exchange only marginally (if at all) reduces the binding strength of haemadin to thrombin. When slow binding inhibition assay was performed at 25°C and physiological ionic strength (0.15 M NaCl) (Figure S2_B), αT was added to solutions of substrate in the presence of increasing concentrations of wild‐type haemadin. From the progress curves of pNA release, the kinetic constants of association (*k*_on_) and dissociation (*k*_off_) of haemadin‐αT complex could be determined as *k*_on_ = 22.53 ± 0.02·10^7^ M^−1^·s^−1^ and *k*_off_ = 4.83 ± 0.02·10^5^ s^−1^, yielding an equilibrium dissociation constant *K*_I_ = *k*_off_/*k*_on_ = 2.14 ± 0.02·10^−13^ M, very close to that estimated from tight binding inhibition analysis (see above) and to that reported by others under similar experimental conditions (Richardson et al., 2002; Strube et al., 1993).

Inhibition data indicate that haemadin has an affinity for the Na^+^‐bound (fast) form, comparable to that of hirudin (*K*_I_ = 0.78 ± 0.02 pM) (Di Cera et al., 1997; Vindigni et al., 1994). However, haemadin binds to the fast form only 7‐fold more tightly than to the Na^+^‐free (slow) form, with a coupling free energy Δ*G*_c_ = Δ*G*_b_(fast) − Δ*G*_b_(slow) = −1.1 kcal/mol. For comparison, full‐length hirudin and its N‐terminal domain 1–47, bind more tightly to the fast form by ~30‐fold (Δ*G*_c_ = −2.1 kcal/mol), compared to the slow form (De Filippis et al., 2005; Di Cera et al., 1997). The higher affinity of haemadin for αT Na^+^‐free (slow) form is likely caused by the presence of the long arginine side chain at position 2 of haemadin, which is expected to facilitate interaction of the inhibitor with Asp189 (at the bottom of αT primary specificity S1 site), to a greater extent in the case of the more closed slow form of the protease than in the case of the fast form, which is already accessible for binding. This interpretation is consistent with our earlier studies on the N‐terminal domain 1–47 of hirudin (De Filippis et al., 2005), showing that replacement of Ser2 with Arg preferentially enhances the affinity for the slow form, yielding a Δ*G*_c_ = −1.1 kcal/mol, identical to that determined for haemadin.

### 2.4 Molecular dissection of haemadin structure and function

**FIGURE 4:**
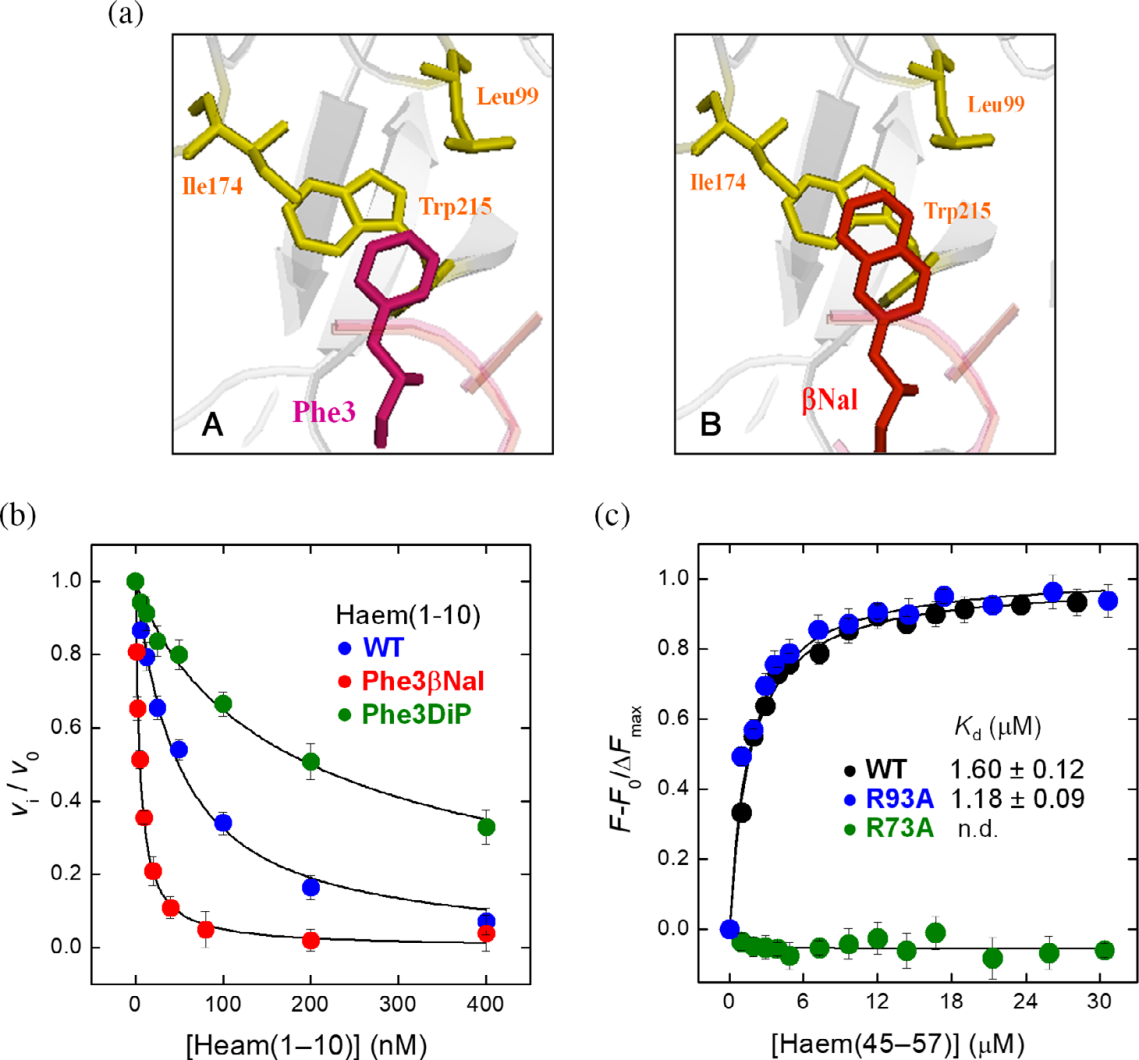
Binding of ψHaem(1–10) and Haem(45–57) peptides to α‐thrombin. (a) Molecular modeling of ψHaem(1–10)Phe3βNal interaction with αT. (Panel A) Close‐up view of the interaction of haemadin Phe3′ with αT, as shown in the haemadin‐αT crystal structure (1e0f.pdb). (Panel B) The modeled β‐naphthyl‐moiety of βNal (red) points toward the apolar S3 site of thrombin (yellow), formed by Leu99, Ile174 and Trp215, and makes a favorable aromatic‐aromatic interaction with Trp215. Modeling was performed using the MOE vs. 15 software. (b) Inhibition of αT amidolytic activity by ψHaem(1–10). αT solutions (250 pM) were incubated with increasing concentrations of pseudo wild type ψHaem(1–10), and its analogues Phe3βNal or Phe3DiP in TBS buffer at 25 ± 0.1°C. The reaction was started by addition of the chromogenic substrate S2238 (50 μM). Values of *v*_i_/*v*_0_ are plotted as a function of inhibitor concentration, where *v*_0_ and *v*_i_ are the steady state velocities of S2238 hydrolysis in the absence or presence of inhibitor. The data points were interpolated with Equation (2) to yield the inhibition constants (*K*_I_) reported in Table 1. Data points are the average of three independent measurements, with errors as ±SD. (c) Binding of Haem(45–57) to recombinant wild‐type and mutant thrombins. The fluorescence increase of a thrombin solution (50 nM) at 334 nm, after excitation at 295 nm, was recorded as a function of peptide concentration (see Section 5). The data points were interpolated with Equation (1) to yield the equilibrium dissociation constants (*K*_D_), as indicated. Data points in binding/inhibition measurements are the average of three independent measurements, with errors as ±SD.

Structural biology data indicate that haemadin is a bivalent αT inhibitor, exploiting its N‐ and C‐terminal regions to interact with the protease active site and exosite II, respectively. Hence, we decided to investigate the αT binding properties of the isolated synthetic peptides corresponding to the sequence 1–10 and 45–57 of haemadin (Figure 2a). The crystal structure of haemadin‐αT complex shows that the inhibitor N‐terminal region is inserted into the active site cleft of αT in a spiral‐like conformation, with the first three amino acids forming a parallel β‐sheet with Ser214‐Gly216 of αT (Richardson et al., 2000) (Figure 4a, panel A). Notably, the amino acids of haemadin are identified with the symbol (′). Arg2′ electrostatically interacts with Asp189 in the S1 site, whereas Ile1′ and Val8′ point toward Tyr60A and Trp60D in the S2 site, and Phe3′ productively interacts with Leu99, Ile174 and Trp215 in the apolar specificity site (S3) of αT. In addition, Met5′ is interspersed between Glu146 (in the γ‐loop) and Glu192 (at the entrance of the active‐site cleft), which in turn favorably interacts with Lys7′ of haemadin. The highly negative C‐terminal tail 45–57 of haemadin covers the electropositive αT exosite II, mainly through charge–charge interactions (Richardson et al., 2000). Based on these considerations, the peptides Haem(1–10) and Haem(45–57) were synthesized by standard SPPS, purified to homogeneity (>98%) by RP‐HPLC, chemically characterized by high‐resolution mass spectrometry (Table S1), and then tested for their αT binding properties. In the case of Haem(1–10), to avoid possible dimerization at Cys10 and oxidation of Met5, a pseudo‐wild type analogue ψHaem(1–10) was produced, in which Cys10 was replaced by Gly and Met5 by nLeu, an oxidation‐resistant methionine isostere.

The affinity of ψHaem(1–10) for αT was determined by measuring the inhibition of αT amidolytic activity on the S2238 substrate, yielding a *K*_I_ = 1.9 ± 0.1 μM (Figure 4b), while the binding of Haem(45–57) was determined by measuring the fluorescence increase of αT at increasing peptide concentrations (Figure 4c), yielding an equilibrium dissociation constant (*K*_D_) of 1.60 ± 0.12 μM. Whereas αT binding properties of the isolated haemadin C‐terminal tail are also shared by hirugen in the hirudin sequence (amino acids 54–65), the relatively high affinity of ψHaem(1–10) for αT is unique to haemadin. In fact, the synthetic peptide VSYTD, corresponding to the N‐terminal segment of hirudin that penetrates into the protease active site (Rydel et al., 1991), lacks any significant inhibition for αT (not shown). Likely, electrostatic interactions of haemadin Arg2′ with Asp189, acting long range, are instrumental to steer the synthetic peptide onto the protease active site and orient other key residues for proper binding to αT.

To possibly enhance the affinity of ψHaem(1–10) for αT, we conducted structure–activity relationship (SAR) studies (De Filippis, Acquasaliente, et al., 2018; De Filippis, Pozzi, et al., 2018) by replacing Phe3′ with natural and non‐natural amino acids, having different side‐chain volume, hydrophobicity, and orientation, and then measuring αT inhibition properties of the resulting synthetic peptides (Figure 4b and Table 1). Our data show that substitution of Phe3′ with less hydrophobic amino acids (i.e., Tyr and Trp) slightly reduced affinity for αT by 1.5‐ or 3.5‐fold. However, the presence of more hydrophobic residues (i.e., Cha, Bip, Dip, αNal and βNal) had very variable, even opposing, effect on αT inhibition, suggesting that other factors, beyond hydrophobicity, play a role in binding to αT. For instance, the decrease of inhibition observed after Phe → Cha exchange, indicates that the aromatic character of the amino acid at position 3 is important in αT binding, while the presence of Bip or Dip, displaying the highest hydrophobic character among the substituting amino acids, left unchanged or reduced the affinity for αT by 4‐fold. It is noteworthy that the replacement of Phe3′ with αNal did not alter inhibition potency, whereas the presence of βNal isomer, sharing with αNal the same size and hydrophobicity, enhanced inhibition by 10‐fold. Modeling studies (Figure 4a, panel B), carried out on the crystal structure of haemadin‐αT complex (Richardson et al., 2000), indicate that βNal orients the additional aromatic ring to make a favorable quadrupole‐quadrupole interaction with Trp215 in the S3 site, whereas αNal side‐chain (like Trp) is oriented toward S2 in a non‐productive manner. SAR studies indicate that, in addition to hydrophobicity, subtle electronic and orientation properties of the amino acid side‐chains at position 3 play a key role in αT inhibition.

**TABLE 1:** Inhibition of αT amidolytic activity by the synthetic wild type and Phe3′ → X analogues of Haem(1–10).^a^

Haemadin (1–10)	*K*_I_ (μM)^b^	ΔΔ*G*_b_ (kcal·mol^−1^)^c^	*r* ^d^	vdW volume (Å^3^)^e^	Log *P* ^f^
WT 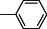	1.9 ± 0.2	–	–	–	–
Met5Nle 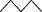	1.9 ± 0.1	–	–	127	2.73
Phe3Tyr 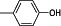	6.6 ± 0.4	0.8	0.3	138	1.97
Phe3Trp 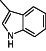	2.9 ± 0.3	0.3	0.7	170	2.33
Phe3Bip 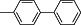	2.3 ± 0.1	0.1	0.8	193	4.73
Phe3DiP 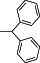	7.4 ± 0.4	0.8	0.3	198	4.80
Phe3Cha 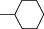	3.8 ± 0.1	0.4	0.5	146	3.88
Phe3αNal 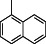	2.3 ± 0.1	0.1	0.8	180	4.00
Phe3βNal 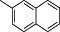	0.19 ± 0.01	−1.4	10	180	4.00

^a^ αT inhibition assays were conducted as detailed in Section 5.

^b^ The values of the equilibrium inhibition constant, *K*_I_, were obtained by analyzing the data according to the tight‐binding model, using Equations (3) and (4).

^c^ ΔΔ*G*_b_ is the difference in free energy change of binding between the analogue (Δ*G*_b_*) and the wild type peptide Haem(1–10), Δ*G*_b_^wt^.

^d^ *r* = *K*_I_^wt^/*K*_I_* is the fold increase in the affinity of the analogues for αT, compared to the wild type peptide.

^e^ van der Waals (vdW) volume.

^f^ Log *P* values of the organic molecules corresponding to the amino acid side chains were taken from Sangster (1989), where *P* is the octanol/water distribution constant.

### 2.5 Molecular mapping of αT regions interacting with full‐length haemadin and haemadin peptides

The regions on αT which are responsible for the binding of full‐length haemadin and isolated haemadin peptides 1–10 and 45–57 were identified by site‐directed mutagenesis, exosite‐specific ligand displacement and HDX‐MS studies.

#### 2.5.1 Full‐length haemadin

##### Binding to αT mutants

Single X → Ala mutants of αT at either exosite I (Phe34Ala, Lys73Ala, Lys110Ala) and II (Arg93Ala, Arg101Ala) were produced and purified to homogeneity as earlier reported (Acquasaliente et al., 2016; Pozzi et al., 2013) (Figure S3) and incubated with haemadin to extract kinetic (*k*_on_ and *k*_off_) and equilibrium (*K*_I_) constants of αT inhibition. The data reported in Table 2 indicate that Ala‐shaving of hydrophobic (Phe) or charged (Arg/Lys) amino acids at exosite‐I does not significantly alter *K*_I_ values, whereas Arg → Ala exchange at position 101 in the exosite‐II causes a 10‐fold decrease of αT inhibition, mainly due to a decrease of *k*_on_, which is consistent with the abrogation of long‐range electrostatic interactions (Schreiber et al., 2006). Interestingly, the same exchange at Arg93 in the exosite II did not alter the affinity of αT for haemadin. These results are in keeping with earlier findings showing that the effect of charge deletion in αT binding to the fibrinogen γ′‐peptide (Alexander et al., 2012), hirudin (Bode et al., 1992), haemadin (Richardson et al., 2000; Strube et al., 1993), and the platelet receptor GpIbα C‐terminal peptide 268–282 (De Cristofaro & De Filippis, 2003) is highly dependent on the mutation site and the nature of charge perturbation (i.e., Ala‐shaving or charge reversal). Charge–charge interactions, in fact, are intrinsically unspecific in nature and the loss of affinity, caused by disruption of a salt bridge in the ionic networks at the protein–protein interface, can be compensated by the formation of novel electrostatic interactions nearby, with minimal (if any) structural/energetic perturbation (Bertonati et al., 2007).

**TABLE 2:** Kinetic constants of the inhibition of recombinant wild type and mutant thrombins by full‐length haemadin.^a^

α‐Thrombin mutants	*k*_on_ (× 10^7^ M^−1^·s^−1^)^b^	*k*_off_ (× 10^−5^ s^−1^)^b^	*K*_I_ (× 10^−13^ M)	*r* ^c^	Exosite^d^
WT	22.53 ± 0.02	4.83 ± 0.02	2.14	–	–
F34A	11.29 ± 0.01	2.95 ± 0.01	2.61	1.2	I
R73A	7.22 ± 0.01	2.45 ± 0.01	3.39	1.6	I
K110A	23.30 ± 0.03	3.06 ± 0.01	1.31	0.6	I
R93A	29.98 ± 0.03	3.68 ± 0.01	1.23	0.6	II
R101A	2.32 ± 0.01	4.99 ± 0.02	21.5	10	II

^a^ αT inhibition assays were conducted as detailed in Section 5.

^b^ The values of the kinetic constants were obtained by analyzing the data according to the slow binding inhibition model, using Equations (5) and (6).

^c^ *r* = *K*_I_*/*K*_i_^wt^ is the fold decrease in the affinity of αT mutants for Haemadin, compared to the wild type protease.

^d^ Localization of X → A mutations.

##### Displacement of PABA and [F]‐hirugen

**FIGURE 5:**
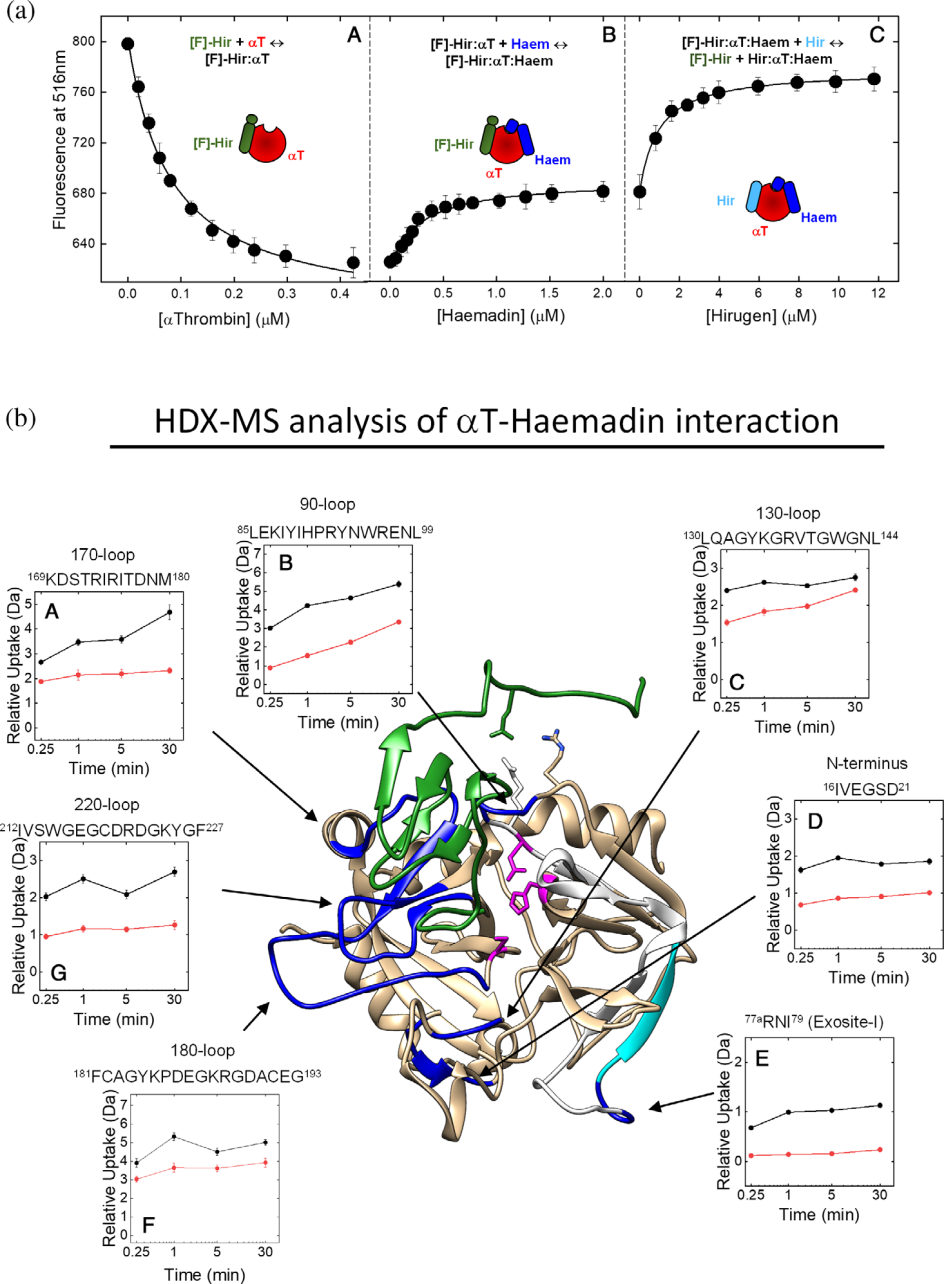
Molecular mapping of haemadin‐αT interaction by spectroscopic and HDX‐MS analysis. (a) Displacement of [F]‐hirugen from exosite I. (Panel A) Fluorescence binding measurements of αT to [F]‐hirugen (60 nM). The data points were interpolated with Equation (2), to yield a *K*_D_ of 68 ± 1 nM. (Panel B) Release of αT‐bound [F]‐hirugen after addition of incremental haemadin concentrations. (Panel C) Displacement of [F]‐hirugen from αT by incremental concentrations of unlabeled hirugen. Binding measurements were carried out at 25 ± 0.1°C in TBS buffer, after sample excitation at 492 nm, and recording the emission intensity at 516 nm. Data points are the average of three independent measurements, with errors as ±SD. (b) Three‐dimensional HDX‐MS difference map of deuterium uptake by αT in the absence and presence of haemadin (green). The values of deuterium uptake were mapped onto the crystal structure of αT‐haemadin complex (1e0f). The regions that are protected from H/D exchange at shorter incubation times (15–60 s) are colored in cyan, whereas the regions that show protection even at longer incubation times (30 min) are in dark blue; the regions that did not display any change in HDX are colored in light orange, while the regions that were not covered in the peptic map (Figure S5) are in white. Catalytic amino acids are shown in magenta. (Panels A–G) Kinetics of relative deuterium uptake of peptic fragments, corresponding to selected regions in αT structure (e.g., exosite I and II, S1 and S3 sites, Na^+^‐binding site), in the absence (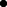) and presence (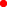) of haemadin. Experimental conditions were as follows: 20°C in 20 mM sodium phosphate in 95:5 D_2_O:H_2_O solution, pD 7.4, containing 150 mM NaCl, at a αT‐haemadin complex concentration of 1.35 μM. Data points in both spectroscopic and HDX‐MS measurements are the average of three independent measurements, with errors as ±SD.

Competition experiments were performed using *p*‐aminobenzamidine (PABA) (Figure S4), as a specific S1 site ligand (Pontarollo et al., 2017), and N^α^‐fluoresceinated hirugen ([F]‐hirugen; Figure 5a), as a specific exosite‐I binder (Pozzi et al., 2013) (see Section 5). The decrease of the relative fluorescence of PABA at increasing inhibitor concentrations indicates that haemadin can interact with αT S1 site and displace PABA, consistent with the observation that haemadin completely abrogates αT amidolytic activity (Figure S2). To study the role of αT exosite I in haemadin binding, we measured the quenching effect of incremental αT concentrations on [F]‐hirugen emission, to yield a *K*_D_ of 68 ± 1 nM identical to that reported in our earlier studies (Acquasaliente et al., 2016) (Figure 5a, panel A). Notably, the affinity of [F]‐hirugen for αT is approximately 20‐fold higher than that of the unlabeled peptide, likely because the fluorescein‐moiety provides and additional binding site for αT. After addition of increasing haemadin concentrations to αT saturated with [F]‐hirugen, about 1/3 of the initial fluorescence decrease was recovered (Figure 5a, panel B), indicating that haemadin was able, to some extent, to displace [F]‐hirugen from exosite I. Al last, the residual [F]‐hirugen, still bound to αT, was displaced by adding increasing concentrations of unlabeled hirugen (Figure 5a, panel C). The partial displacement of [F]‐hirugen might be explained considering a promiscuous binding of haemadin C‐terminal tail between exosite I and II or, alternatively, assuming that binding of haemadin to exosite II promotes displacement of [F]‐hirugen from exosite‐I through a negative allosteric effect, which has been earlier proposed to operate between αT exosites (Chen et al., 2017; Petrera et al., 2009).

##### HDX‐MS analysis

Direct binding of haemadin to αT in solution was studied by HDX‐MS, an emerging powerful analytical method in structural biology, useful for investigating protein conformation and dynamics and molecular recognition (Engen & Komives, 2020; Masson et al., 2019; Sowole & Konermann, 2014). HDX‐MS basically exploits the intrinsic propensity of backbone amide hydrogens at exposed/flexible sites to exchange more rapidly with deuterium than those hydrogens that are buried in the protein interior or at the ligand‐protein interface and therefore will exchange much more slowly (Masson et al., 2019; Sowole & Konermann, 2014). HDX can be monitored by recording the time‐dependent mass increase of short fragments, generated after proteolysis with pepsin, allowing the study of protein dynamics and ligand‐protein interaction with a spatial resolution of 3–6 amino acids (Masson et al., 2019).

HDX‐MS analysis of αT was carried out at 20°C in PBS, pH 7.4, at 0.15 or 0.4 M NaCl, before and after addition of saturating haemadin concentrations. A coverage of 92% in the αT sequence was obtained, allowing us to generate a bi‐dimensional heatmap of deuterium uptake for each peptide at increasing incubation times, from 15 s to 30 min, in the absence and presence of haemadin (Figure S5). The regions that were not covered by HDX analysis, due to the lack of suitable peptic fragments, are essentially located in the 60‐loop, shaping the S2 site, and the ^100^Asp‐Leu^105^ segment, encompassing the catalytic Asp^102^. Notably, the other two catalytic amino acids (i.e., His^57^ and Ser^195^) and Asp194, which in mature αT forms a high‐energy salt bridge with Ile16 N‐terminus (Bode et al., 1992), do not undergo H/D exchange even at longer incubation times and higher temperatures (e.g., 16 h at 37°C). These results reflect the intrinsic stability of the catalytic pocket and the key amino acids involved in αT maturation. As Asn‐60g in the insertion 60‐loop undergoes variable N‐glycosylation in natural αT, we could not identify any suitable peptic fragment covering the 60‐loop region, thus loosing information on the effect that haemadin binding might have on the conformation of the S2 site.

To more easily identify the regions that are conformationally altered upon inhibitor binding, a difference three‐dimensional heatmap of deuterium uptake was generated on the crystallographic structure of haemadin‐αT complex (Richardson et al., 2000) (Figure 5b). Our data indicate that the binding of haemadin induces shielding/ordering of well‐defined αT regions, with a notable decrease in the kinetics of deuterium uptake in the insertion loop regions shaping the S1 (^181^Phe‐Glu^192^) and S3 (^95^Asn‐Leu^99^, ^171^Ser‐Met^180^ and ^215^Trp‐Phe^227^) sites, and the Na^+^‐binding site nearby (i.e., 180‐loop and 220‐loop). Interestingly, rigidification of these regions is transmitted long‐range, beyond the inhibitor‐protease contact surface, to the flanking regions comprising the segment ^16^Ile‐Glu^23^ in the B‐chain N‐terminal segment in the activation domain and the sequence ^142^Gly‐Leu^144^ in the γ‐loop. Even some parts of the 70‐loop and the regions ^77^Glu‐Leu^85^ and ^81^Lys‐His^91^ in the exosite I become more protected from H/D exchange when αT is bound to haemadin, whereas H/D exchange is not significantly altered for the amino acids that contribute to exosite II.

#### 2.5.2 ψHaem(1–10)Phe3βNal

##### HDX‐MS analysis

**FIGURE 6:**
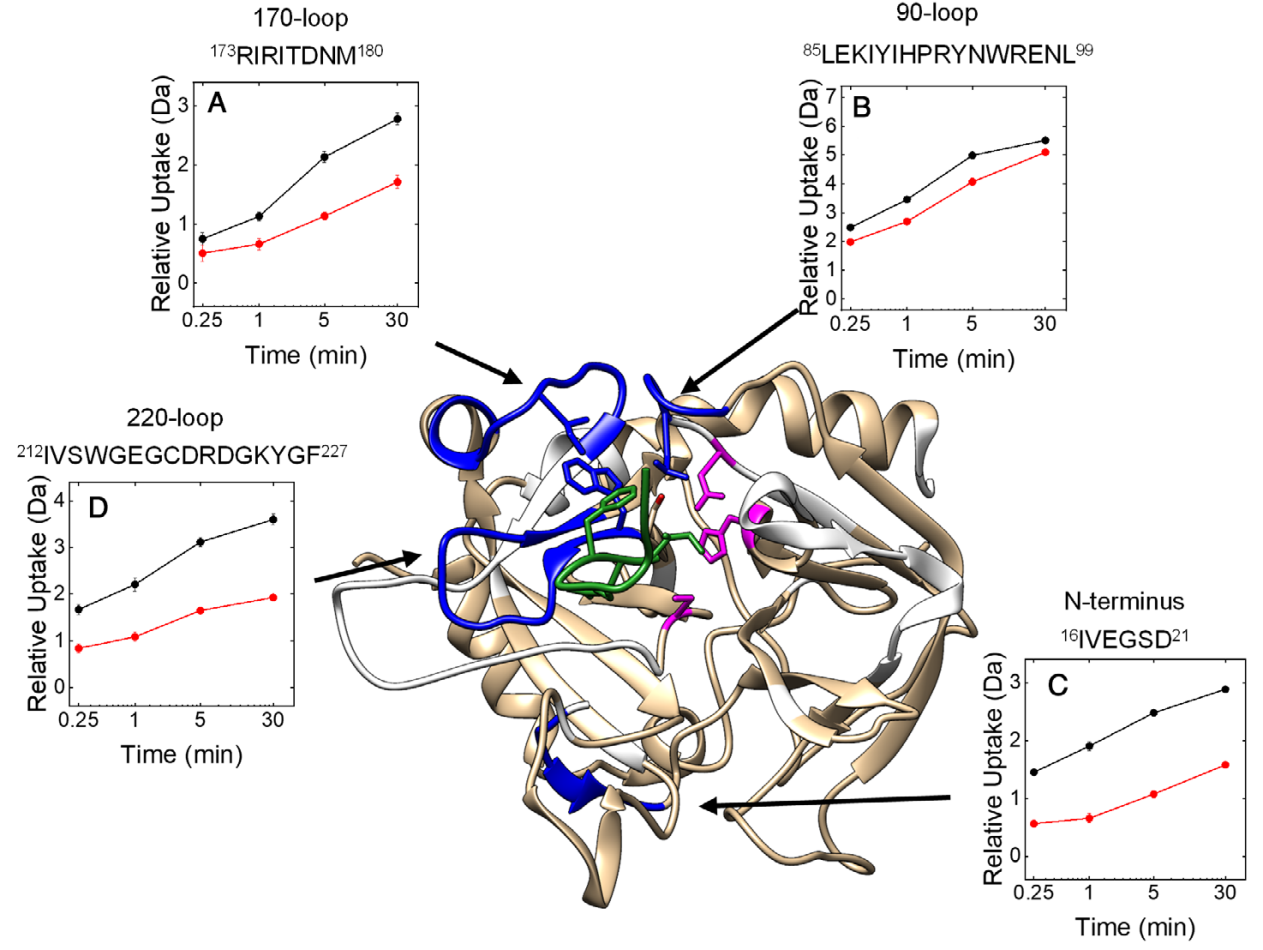
Molecular mapping of ψHaem(1–10)Phe3βNal binding to αT by HDX‐MS analysis. Three‐dimensional HDX‐MS difference in deuterium uptake by αT in the absence and presence of ψHaem(1–10)Phe3βNal (green). The deuterium uptake values were mapped onto the modeled structure of ψHaem(1–10)Phe3βNal bound to αT. The regions that are always protected from H/D exchange in the 15 s to 30 min time range are shown in dark blue; the regions that did not display any change in HDX are colored in light orange, while the regions that were not covered in the peptic map (Figure S6) are in white. The catalytic amino acids are in magenta. (Panels A–D) Kinetics of relative deuterium uptake of peptic fragments, corresponding to selected regions in αT structure (e.g., exosite I and II, S1 and S3 sites, Na^+^‐binding site), in the absence (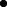) and presence (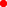) of haemadin. Experimental conditions were as follows: 20°C in 20 mM sodium phosphate in 95:5 D_2_O:H_2_O solution, pD 7.4, containing 150 mM NaCl, at a αT‐haemadin complex concentration of 1.35 μM. HDX‐MS measurements were conducted in triplicate with error bars as ±SD.

As expected from enzyme inhibition assays, HDX‐MS analysis of the more potent ψHaem(1–10)Phe3βNal analogue binding to αT indicates remarkable protection from H/D exchange in the S1 (^181^Phe‐Glu^192^) and S3 (^96^Trp‐Leu^99^, ^171^Ser‐Met^180^ and ^215^Trp‐Phe^227^) sites (Figures 6 and S6), together with the Na^+^‐binding site nearby, as already observed with full‐length haemadin. Even with ψHaem(1–10)Phe3βNal, ordering was also observed in the flanking region corresponding to the N‐terminal segment ^16^Ile‐Glu^23^ of the B‐chain. However, in contrast to haemadin, ψHaem(1–10)Phe3βNal does not appear to increase protection in the γ‐loop nor in the αT exosite I.

#### 2.5.3 Haem(45–57)

##### Binding to charge‐deletion αT mutants

The affinity of the synthetic peptide Haem(45–57) for charge‐deleted mutants at αT exosite I (Arg73Ala) and exosite II (Arg93A) was measured by recording the increase of αT fluorescence at 334 nm as a function of peptide concentration and compared to that measured for wild‐type αT (Figure 4c). Our data indicate that ablation of the positive charge at exosite I results in a dramatic drop of affinity, whereas the same mutation at exosite II leaves the affinity unchanged. These results show that the structural integrity of exosite I is important for binding and suggest that Haem(45–57) preferentially binds to αT exosite I.

##### Displacement of [F]‐hirugen

**FIGURE 7:**
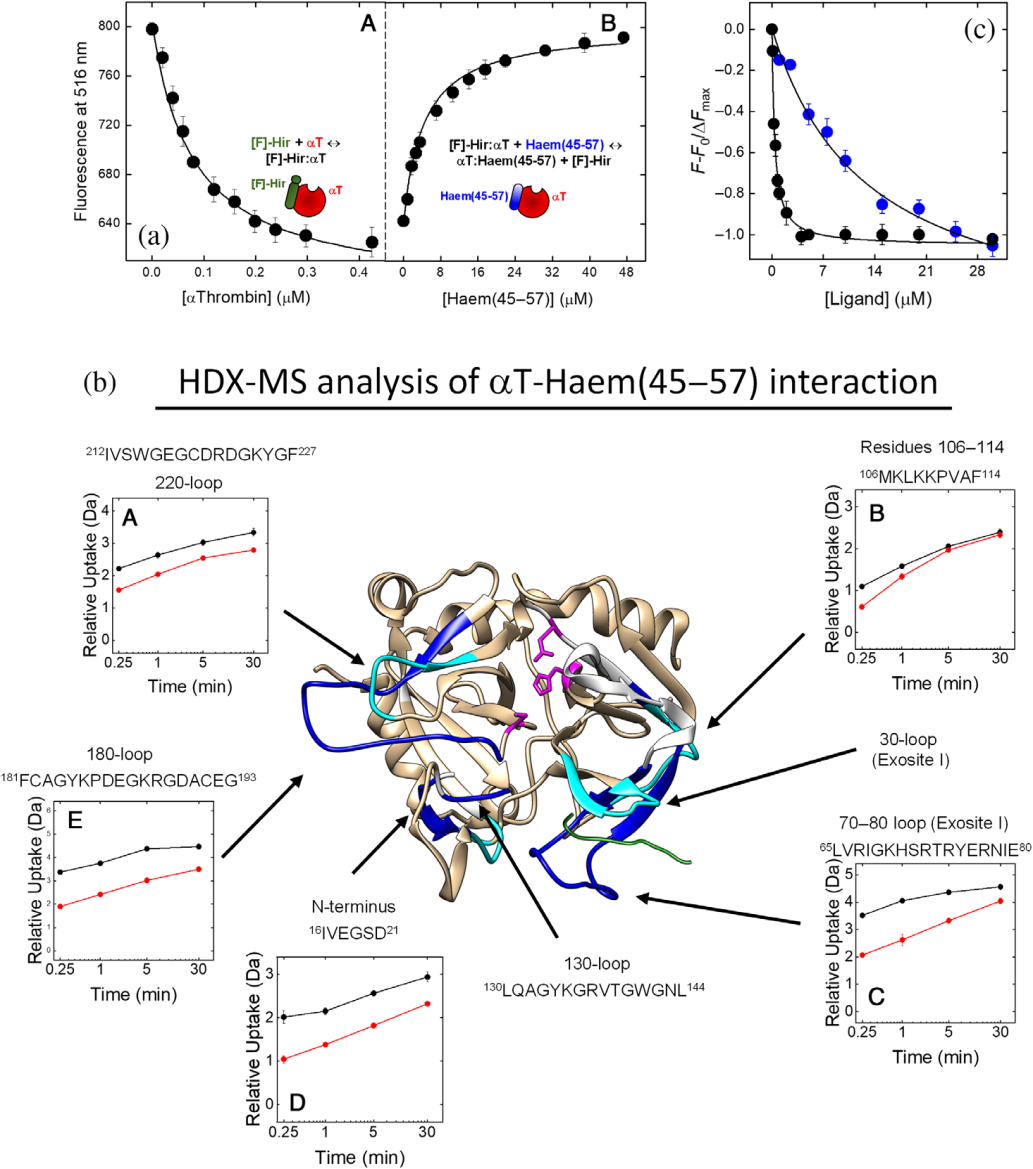
Molecular mapping of Haem(45–57)‐αT interaction by spectroscopic and HDX‐MS analysis. (a) Displacement of [F]‐hirugen from exosite‐I. (Panel A) Fluorescence binding measurements of αT to [F]‐hirugen (60 nM). The data points were interpolated with Equation (2), to yield a *K*_D_ of 65 ± 2 nM. (Panel B) Quantitative displacement of [F]‐hirugen bound to αT after the addition of incremental haemadin concentrations. Binding measurements were carried out at 25 ± 0.1°C in TBS buffer, after sample excitation at 492 nm, and recording the emission intensity at 516 nm. Data points are the average of three independent measurements, with errors as ±SD. (b) Three‐dimensional HDX‐MS difference map of deuterium uptake by αT in the absence and presence of haemadin (green). The values of deuterium uptake were mapped onto the crystal structure of αT‐haemadin complex (1e0f). The regions that are protected from H/D exchange at shorter incubation times (15–60 s) are colored in cyan, whereas the region that show protection even at longer incubation times (30 min) are in dark blue; the regions that did not display any change in HDX are colored in light orange, while the regions that were not covered in the peptic map (Figure S7) are in white. Catalytic amino acids are shown in magenta. (Panels A–E) Kinetics of relative deuterium uptake of peptic fragments, corresponding to selected regions in αT structure (e.g., exosite I and II, S1 and S3 sites, Na^+^‐binding site), in the absence (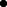) and presence (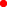) of haemadin. Experimental conditions were as follows: 20°C in 20 mM sodium phosphate in 95:5 D_2_O:H_2_O solution, pD 7.4, containing 150 mM NaCl, at a αT‐haemadin complex concentration of 1.35 μM. (c) Direct binding of [F]‐Haem(45–57) to isolated αT S195A (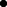) or to the preformed S195A‐hirudin complex (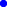). Incremental concentrations of the S195A or S195A‐hirudin complex were added to a solution of [F]‐Haem(45–57) (60 nM). The samples were excited at 492 nm and the emission intensity was recorded at 516 nm. The data points were interpolated with Equation (1) to yield *K*_D_ values of 0.35 ± 0.05 μM and 12.8 ± 1.5 μM for the binding of [F]‐Haem(45–57) to the free αT and αT‐hirudin complex, respectively. Data points in both spectroscopic and HDX‐MS measurements are the average of three independent measurements, with errors as ±SD.

Using the same procedure previously exploited with full‐length haemadin, we checked whether Haem(45–57) was able to displace [F]‐hirugen from αT exosite I. The data in Figure 7a, clearly indicate that this is actually the case, as addition of Haem(45–57) to a αT solution saturated with [F]‐hirugen results in the complete recovery of the unbound [F]‐hirugen fluorescence intensity. This is a further, stringent indication that, contrary to the parent intact molecule, the isolated haemadin C‐terminal tail binds to αT exosite I.

##### HDX‐MS analysis

The results of HDX‐MS analysis of Haem(57–57) binding to αT (Figures 7b and S7) were compared with those obtained for a specific exosite‐I binder like hirugen (Figure S8) and for a bivalent active site and exosite‐I strong binder like hirudin (Figure S9). Our analysis shows strong H/D protection in the 70‐loop (^65^Leu‐Leu^85^) and, to a lesser extent, in the 30‐loop (^34^Phe‐Leu^40^), where both regions shape the exosite I cleft. Protection at short incubation times also involves the 110‐loop (^107^Lys‐Phe^114^) at the periphery of exosite I. It is noteworthy that, upon peptide binding, ordering is transmitted long‐range to the 180‐loop (^182^Cys‐Gly^193^) and 220‐loop (^215^Trp‐Phe^227^), forming the S1 site, the Na^+^‐binding site, and part of the S3 site. As observed with full‐length haemadin and ψHaem(1–10)Phe3βNal binding to αT active site, even the segment ^16^Ile‐Glu^23^ in the B‐chain N‐terminal end and the sequence ^142^Gly‐Leu^144^ in the γ‐loop become more protected. Importantly, the H/D exchange profile induced by Haem(45–57) closely resembles that of αT bound to hirugen and hirudin.

##### Binding of [F]‐Haem(45–57) to hirudin‐saturated αT

The results of mutagenesis, spectroscopic and HDX‐MS studies, reported above, converge to indicate that isolated Haem(45–57) binds to αT exosite I. At this point, we asked whether this peptide has some intrinsic affinity for exosite II, which is the cognate binding site of the C‐terminal tail in intact haemadin. To possibly answer this question, we synthesized the N^α^‐fluoresceinated analogue of the peptide 45–57, [F]‐Haem(45–57), and measured the decrease in fluorescence intensity of αT‐hirudin complex at increasing peptide concentrations, where both the active site and exosite I in the pre‐formed complex are blocked by hirudin tight binding. From the fluorescence binding data in Figure 7c, a *K*_D_ of 0.35 ± 0.05 μM was estimated for the binding of [F]‐Haem(45–57) to free αT, whereas a >35‐fold lower affinity (*K*_D_ = 12.8 ± 3.2 μM) was estimated for the binding to the complex formed by the catalytically inactive αT S195A mutant and hirudin.

Altogether, these results indicate that the haemadin C‐terminal segment 45–57 has an intrinsic binding preferentiality for αT exosite I when it is isolated in solution, whereas it binds (with much lower affinity) to exosite II when exosite I is not available (i.e., after saturation with the stronger binder hirudin) or when the 45–57 sequence is embedded in the full‐length haemadin, which orients the negative tail toward exosite II.

### 2.6 Design and synthesis of haemanorm

**FIGURE 8:**
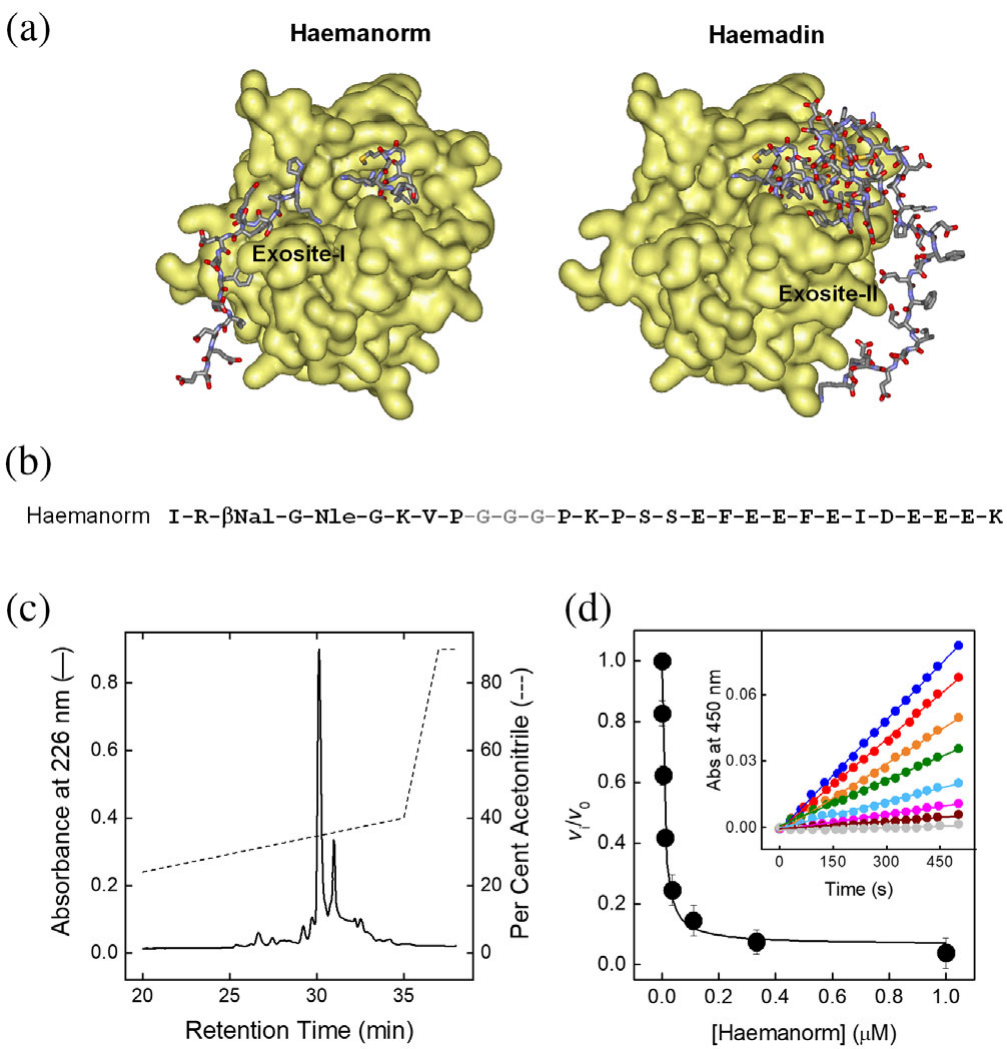
Design, synthesis and αT inhibition properties of haemanorm. (a, left panel) Design of haemanorm. Schematic representation of the interaction of haemadin sequence 1–9 to αT active site and haemadin sequence 41–57 to the protease exosite I. The van der Waals surface of αT is colored light gold, while the haemadin segments are in stick (color coded: Nitrogen, blue; Oxygen, red; Carbon, gray). The image is based on the coordinates extracted from the crystallographic structure of haemadin–αT complex (1e0f.pdb), showing that the αT molecule (chains A and D) in the asymmetric cell unit binds at the active site (and exosite II) of a haemadin molecule (chain I, sequence 1–9) and at the exosite I to another Haemadin molecule (chain J, sequence 41–57). (Right panel). For comparison, a schematic representation of full‐length haemadin binding to αT, showing the bivalent binding at the active site and exosite II, is also reported. (b) Amino acid sequence of haemanorm. The sequence of haemadin was simplified by inserting a glycine‐linker between the sequences 1–9 and 41–57 of haemadin. (c) RP‐HPLC analysis of the crude synthetic haemanorm, after resin cleavage and side‐chain deprotection. An aliquot (10 μg) of the crude peptide was injected into a Zorbax C18 analytical column (4.6 mm × 150 mm), eluted with a linear acetonitrile gradient (‐ ‐ ‐) in 0.1% (v/v) aqueous TFA, from 5% to 50% in 40 min, at a flow rate of 0.8 mL/min. The peptide material eluted with the major peak was collected and analyzed by HR‐MS, yielding a mass value in agreement with the amino acid composition within 10 ppm mass accuracy (see Table S1). (d) Inhibition of αT amidolytic activity. αT solutions (500 pM) were incubated with increasing concentration of haemanorm and added with S2238 (100 μM). The values of *v*_i_/*v*_0_, were plotted as a function of the inhibitor, as indicated. The data were analyzed using the tight‐binding inhibition model (Equations 3 and 4), to yield a *K*_Ι_ value of 0.80 ± 0.01 nM. Enzyme inhibition assays were conducted at 25 ± 0.1°C in TBS, pH 7.4. Data points are the average of three independent measurements, with error bars as ±SD.

The findings that isolated ψHaem(1–10) and Haem(45–57) bind to αT active site and exosite I, respectively, with affinities in the low micromolar range, prompted us to design a peptide containing both the N‐ and C‐terminal haemadin segments joined by a flexible peptide linker (Figure 8a,b). With this in mind, we synthesized and characterized haemanorm, a 29‐amino acid peptide containing: (i) the sequence 1–9 of haemadin carrying the isosteric substitution Met5′ → nLeu, (ii) the Phe3′ → βNal replacement, which has been shown to improve T inhibition, (iii) a flexible linker of three glycines, and (iv) the sequence 41–57 of haemadin, starting from the ^41^Pro‐Lys‐Pro^43^ motif, which is expected to rigidify the C‐terminal tail of haemadin for αT binding. The crude peptide was purified by RP‐HPLC and chemically characterized by high‐resolution MS (Figure 8c). The affinity of haemanorm for αT was determined by enzyme inhibition measurements at 25°C in TBS, pH 7.4, containing 0.15 M NaCl. Data were analyzed according to the tight‐binding inhibition model, allowing us to estimate a *K*_I_ value as low as 0.80 ± 0.01 nM and a Δ*G*_b_ = RT∙ln*K*_I_ = −12.4 kcal/mol. Hence, haemanorm was found to inhibit αT with an affinity 2.000‐ or 2.370‐fold higher than that measured for isolated Haem(45–57) (*K*_I_ = 1.6 ± 0.1 μM) or ψHaem(1–10) (*K*_I_ = 1.9 ± 0.1 μM) peptides (Figure 8d). Furthermore, RP‐HPLC analysis (not shown) of haemanorm‐αT reaction mixture (1–12 h incubation) indicates that haemanorm is fully resistant to proteolysis by αT at the Arg2‐βNal bond, which is a potential cleavage site for the protease.

### 2.7 Molecular mapping of haemanorm binding to αT

**FIGURE 9:**
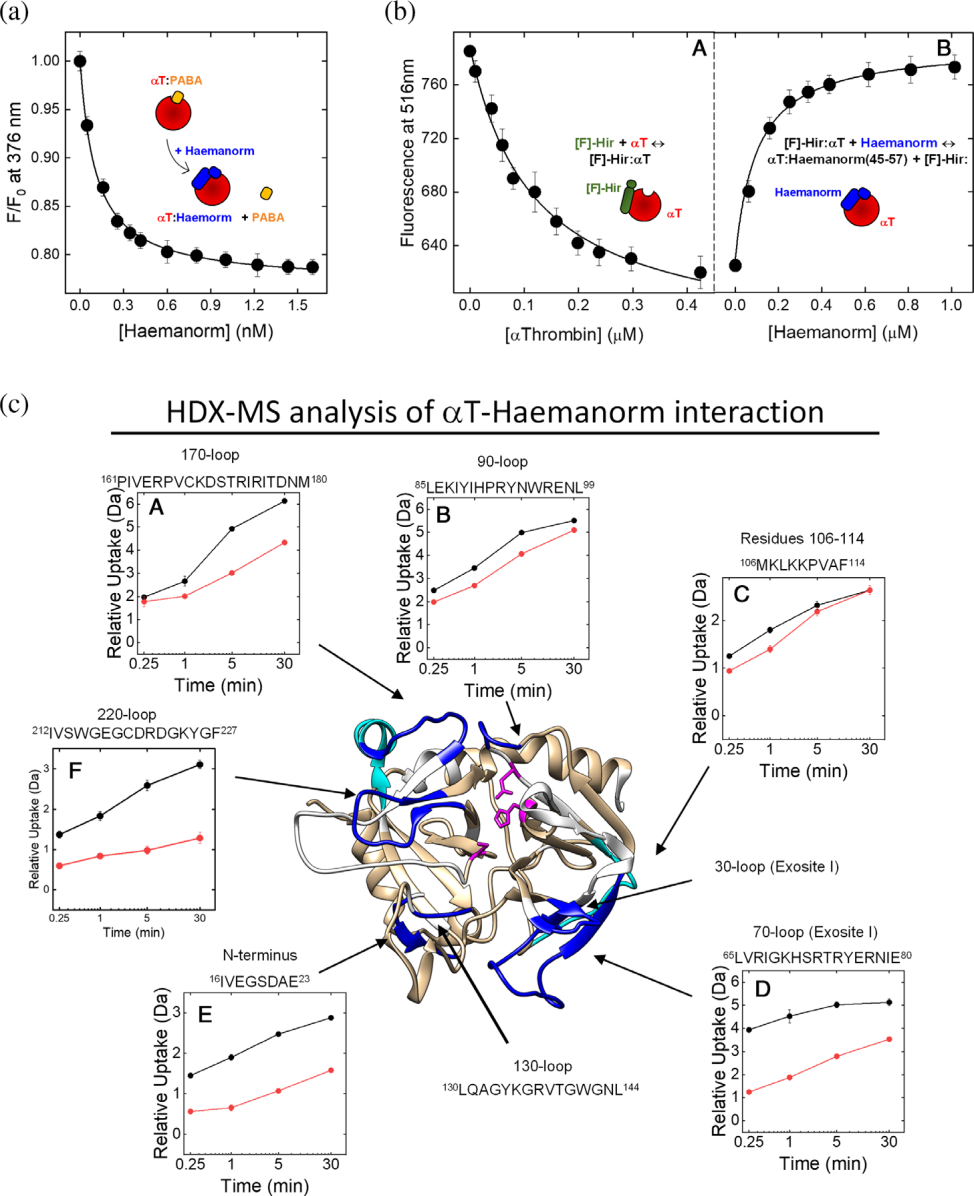
Molecular mapping of haemanorm‐αT interaction by spectroscopic and HDX‐MS analysis. (a) Displacement of PABA from the primary specificity S1 site. A solution of αT (50 nM) in TBS was first incubated at 25 ± 0.1°C with a saturating concentration of PABA (500 nM) and then added with increasing haemanorm concentrations. Samples were excited at 335 nm and the release of PABA was monitored by recording the decrease of fluorescence signal at 376 nm, after base line subtraction. Data are reported as the *F*/*F*_0_ ratio, where *F*_0_ and *F* are the emission intensities of PABA in the absence and presence of haemanorm. (b) Displacement of [F]‐hirugen from exosite‐I. (Panel A) Fluorescence binding measurements of αT binding to [F]‐hirugen (60 nM). The data points were interpolated with Equation (2), to yield a *K*_D_ of 65 ± 2 nM. (Panel B) Quantitative displacement of [F]‐hirugen bound to αT, after addition of incremental haemanorm concentrations. Binding measurements were carried out at 25 ± 0.1°C in TBS buffer, after sample excitation at 492 nm, and recording the emission intensity at 516 nm. (c) Three‐dimensional HDX‐MS difference map of deuterium uptake by αT in the absence and presence of haemanorm. The values of deuterium uptake were mapped onto the crystal structure of αT‐haemadin complex (1e0f). The regions that are protected from H/D exchange at shorter incubation times (15–60 s) are colored in cyan, whereas the region that show protection even at longer incubation times (30 min) are in dark blue; the regions that displayed any change in HDX are colored in light orange, while the regions that were not covered in the peptic map (Figure S10) are in white. Active site amino acid (H57, D102, S195) are shown in magenta, (Panels A–F) Kinetics of relative deuterium uptake of peptic fragments, corresponding to selected regions in αT structure (e.g., exosite I and II, S1 and S3 sites, Na^+^‐binding site), in the absence (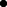) and presence (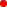) of haemanorm. Experimental conditions were as follows: 20°C in 20 mM sodium phosphate in 95:5 D_2_O:H_2_O solution, pD 7.4, containing 150 mM NaCl, at a αT‐haemanorm complex concentration of 1.35 μM. Data points in both spectroscopic and HDX‐MS measurements are the average of three independent measurements, with errors as ±SD.

As reported above for haemadin and isolated haemadin peptides, the binding regions for haemanorm on αT structure were identified by displacement experiments and HDX‐MS analysis. Notably, haemanorm was able to quantitatively displace PABA from the S1 site (Figure 9a) and [F]‐hirugen from exosite I (Figure 9b), thus providing solid experimental evidence for its bivalent mechanism of binding to αT. However, conclusive indications on the αT regions that are involved in haemanorm binding came from HDX‐MS measurements (Figures 9c and S10). Protection from H/D exchange was observed in the very same regions that in αT become protected after binding to isolated ψHaem(1–10)Phe3βNal (Figure 6) or Haem(45–57) (Figure 7) and to hirugen (Figure S8) or hirudin (Figure S9), taken as benchmarks of monovalent and bivalent exosite‐I ligands, respectively. As for ψHaem(1–10)Phe3βNal binding, loop regions encompassing the S1 and S3 (96Trp‐Leu99, 169Lys‐Met180, 215Trp‐Phe227) sites become more protected, along with the flanking regions ^16^Ile‐Glu^23^ in the N‐terminal segment of the B‐chain and ^142^Gly‐Leu^144^ in the γ‐loop. Likewise, moderate to strong protection is observed at ^34^Phe‐Leu^40^, ^66^Val‐Leu^85^ and ^107^Lys‐Phe^114^ sequences in the exosite I. Hence, HDX data provide clear‐cut, direct evidence for the bivalent nature of haemanorm binding to αT active site and exosite I.

## 3 DISCUSSION

During the last 50 years, the pharmaceutical repertoire for anticoagulant therapy has been essentially limited to heparin and dicoumarol derivatives (Hirsh et al., 2019). While exploiting different mechanisms, these anticoagulants may be considered “indirect” inhibitors of αT generation/function, and for this reason their use in therapy is often associated with major side effects and requires careful monitoring (Hirsh et al., 2019; Wardrop & Keeling, 2008). The relevance of pharmacological applications of hirudin and its analogues have stimulated the resurgence of pharmaceutical interest in natural products isolated from hematophagous organisms for developing new anticoagulant drugs selectively targeting/inhibiting αT or other coagulation factors (Corral‐Rodríguez et al., 2010; De Filippis, Acquasaliente, et al., 2018; De Filippis, Pozzi, et al., 2018; Huntington, 2014; Lombardi et al., 1999). Among these inhibitors, haemadin from *Haemadipsa sylvestris* can be regarded as a promising alternative to hirudin and bivalirudin (Kostromina et al., 2021; Richardson et al., 2000; Strube et al., 1993).

### 3.1 Chemical synthesis and characterization of haemadins

The results reported in this work provide evidence that stepwise chemical synthesis, followed by in vitro disulfide oxidative refolding and RP‐HPLC purification, is a fast and versatile method to produce in relatively high yields large amounts of fully active wild‐type haemadin preparations with pharmaceutical purity (>98%). Chemical synthesis also allowed incorporation of noncoded oxidation resistant amino acids (i.e., Met5 → nLeu) to improve the chemical stability of the protein without appreciably losing activity.

Our data indicate that the bottleneck of haemadin production is the oxidative disulfide refolding step. After 24‐h folding reaction, the chromatographic yield of haemadin with native disulfide topology (N‐Haem) was at most ~60% (Figure 2b), lower than that of full‐length hirudin (>85%) (Chatrenet & Chang, 1993) or its N‐terminal domain 1–47 (>95%) (De Filippis et al., 1995; De Filippis et al., 1998), with which haemadin shares identical SS bond pairing and a similar number of prolines. To possibly explain these differences, it should be considered that in the unfolded protein approximately 80% and 20% of prolines are in the *trans* or *cis* conformation, respectively, and that *cis ↔ trans* proline isomerization is required for correct protein folding. This reduces the fraction of correctly isomerized polypeptide chains that in the starting (unfolded) ensemble drives folding to the proper *trans* or *cis* proline isomers found in the native state (Wedemeyer et al., 2002). Because proline isomerization is much slower than (conformational and disulfide oxidative) renaturation, incorrectly folded polypeptide chains with non‐native disulfides can be formed and trapped during the folding process (Welker et al., 2001). On these grounds, it is expected that the role of prolines is even more relevant when they are comprised in the disulfide folding nucleus. In the case of haemadin, three of the four prolines are in the *trans* conformation, whereas Pro43 displays a *cis* conformation (Richardson et al., 2000). More importantly, these prolines are all located within or very close to the compact, disulfide crosslinked polypeptide chain (i.e., from Cys10 to Cys37) (Strube et al., 1993) (Figure 2a). At variance, the three prolines in the hirudin sequence are all in the *trans* conformation and located in the highly flexible C‐terminal tail, far from the disulfide knotted N‐terminal domain (Folkers et al., 1989; Rydel et al., 1991). These considerations might explain on simple grounds the lower efficiency of haemadin folding, compared to that of hirudin.

The unusual resistance of haemadin and hirudin to thermal denaturation (Figure 3d) reflects a general property of small‐sized, disulfide bridged globular proteins (Alexander et al., 1992; Myers et al., 1995; Privalov & Gill, 1988). The conserved three disulfides present in both haemadin and hirudin are indeed expected to contribute to their stability by preferentially reducing the main‐chain entropy of the denatured state and, consequently, the conformational entropy change of denaturation (Δ*S*_D_) (Harrison & Sternberg, 1994; Pace & Grimsley, 1988). Other possible stabilizing factors for haemadin involve a higher content of prolines and uncompensated surface charges, compared to that of hirudin. In fact, proline is known to stabilize proteins by reducing Δ*S*_D_ value (Matthews et al., 1987) and even by restricting the number of possible local conformations of the protein backbone in the native state (Fontana et al., 1993; Fontana et al., 1998). It is noteworthy that the relative abundance of proline in the folded N‐terminal domain (amino acids 1–43) of haemadin is approximately two‐fold higher (9.3%) than that found in the corresponding hirudin domain (amino acids 1–47) (4.2%) (Figure 2a) and in globular proteins (5.1%) (https://www.proteinatlas.org). Furthermore, electrostatic repulsion between equally charged residues on protein surfaces have been shown to preferentially destabilize the thermally denatured state, compared to the native state, thus opposing protein denaturation (Perl et al., 2000). This effect is expected to be even more pronounced when the polypeptide chain in the denatured state still retains some compact structure, as in the case of disulfide cross‐linked haemadin (*T*_m_ ~ 85°C) and hirudin (*T*_m_ ~ 63°C) (Figure 3d). Interestingly, haemadin contains significantly more charged amino acids (19) in its sequence than hirudin (13) (Figure 2a) and none of these is compensated by intramolecular salt‐bridges or hydrogen bonds (Folkers et al., 1989; Richardson et al., 2000; Rydel et al., 1991). Altogether, the observations reported above suggest that these small protein inhibitors might have achieved resistance to thermal denaturation by variably optimizing those properties (i.e., chain length, SS bonds and proline content, and electrostatic interactions) that oppose denaturation by mainly destabilizing the denatured state rather than stabilizing the native state.

### 3.2 Structural and functional dissection of haemadin binding to αT

Earlier mutagenesis studies on αT showed that charge‐reversal mutations (i.e., Lys/Arg → Glu) in the protease exosite II reduced the affinity for haemadin by 10‐fold, whereas the same substitutions at exosite I had only limited effect, suggesting that exosite II is primarily involved in the interaction with haemadin (Richardson et al., 2000; Strube et al., 1993). Nevertheless, haemadin binding was not inhibited by heparin, a sulphated glycosaminoglycan specifically binding to αT exosite II (Richardson et al., 2000; Strube et al., 1993), highlighting the possibility that haemadin can also bind to exosite I. Unfortunately, the crystallographic structure of haemadin‐αT complex did not conclusively clarify the ambiguity of biochemical data. The available crystal structure of the complex, in fact, shows that the inhibitor N‐terminal region always targets the protease active site in all the three molecular complexes present in the asymmetric unit cell, whereas in one of the complexes the C‐terminal segment 45–57 is not bound to the cognate αΤ molecule but to the exosite I of another neighboring molecule in the asymmetric unit (Richardson et al., 2000). Whether these binding properties arise from crystallization artifacts (e.g., crystal contacts or conformational selection by the crystal) or, instead, reflect a “real” promiscuity of haemadin binding to αT in solution, has not yet been firmly established.

To fill this knowledge gap, we decided to map the regions on αT which are responsible for the binding of full‐length haemadin and isolated haemadin peptides 1–10 and 45–57 using site‐directed mutagenesis, exosite‐specific ligand displacement and HDX‐MS studies. The picture that emerges from these studies shows that the N‐terminal segment of haemadin targets αT active‐site cleft either in the intact protein and in the isolated peptide ψHaem(1–10). Indeed, both inhibitors compete with the chromogenic substrate for the binding to the enzyme active site (Figure S2, Tables 1 and 2), displace PABA from the S1 primary specificity site (Figure S4), and induce protection from H/D exchange of similar regions in αT structure (Figures 5b and 6). Conversely, the isolated C‐terminal peptide Haem(45–57) displays binding properties which are different from those it unfolds when embedded in the intact haemadin, as will be discussed in the following. In fact, perturbation of exosite I by Arg73Ala mutation abrogates affinity of Haem(45–57) for αT (Figure 4b), whereas the same Arg → Ala mutation at position 93 in the exosite II does not affect binding of Haem(45–57), suggesting that the integrity of exosite I (but not of exosite II) is required for peptide binding. Furthermore, Haem(45–57) quantitatively displaced [F]‐hirugen from αT exosite I (Figure 7a) and induced protection from H/D exchange of the 30‐ and 70‐loop regions in the exosite I (Figure 7b), very similar to that observed after binding of hirugen (Figure S8) or full‐length hirudin (Figure S9). Nevertheless, Haem(45–57) was found to interact with exosite II (although with a >35‐fold lower affinity compared to exosite‐I binding, *K*_d_ = 12.8 μM) in the αT‐hirudin complex (Figure 7c). The affinity of free Haem(45–57) for αT exosite II thus estimated is in the same order of magnitude as that extrapolated theoretically for the binding of the C‐terminal tail 41–57 in the intact inhibitor (*K*_d_ ~ 50 μM), starting from the *K*_i_ values of full‐length haemadin (*K*_i_ = 0.25 pM) and the N‐terminal domain 1–40 (*K*_i_ = 5 nM) experimentally determined (Richardson et al., 2002) and assuming that the N‐ and C‐terminal regions bind independently to αT with an additive effect on the affinity for αT: *K*_d (41–57)_ = *K*_I (1–57)_/*K*_I (1–40)_. Altogether, these data concurrently indicate that Haem(45–57) displays intrinsic binding preferentiality for exosite I and that it can even bind to exosite II, but with lower affinity and only when exosite I is not available.

In the case of intact haemadin, Arg101Ala mutation at exosite II results in a 10‐fold drop in the affinity for αT, whereas perturbation of exosite I at different sites (including Arg73Ala substitution) has very limited (if any) effect (see Table 2). These results are in keeping with earlier mutagenesis data (Richardson et al., 2002; Strube et al., 1993) and seem to confirm that, conversely to what observed with isolated Haem(45–57), exosite II is the preferred binding site for the C‐terminal region in full‐length haemadin, likely because the rigid stereochemistry of the haemadin N‐terminal domain interaction with αT active site obligatorily drives the highly flexible C‐terminal tail toward exosite II, thus impairing haemadin binding to the exosite I of the cognate αT molecule. However, our data also indicate that haemadin can displace (albeit non‐quantitatively) [F]‐hirugen from exosite I (Figure 5a) and that its binding to αT significantly protects some regions of exosite I from H/D exchange, without appreciably altering the kinetics and extent of deuterium uptake at exosite II (Figure 5b). Regarding the latter result, the relative insensitivity of HDX‐MS to protein–protein interaction is often observed in low‐affinity systems (*K*_d_ ≫ 1 μM), when the population of bound proteins during H/D exchange is low, or if complex formation primarily involves side‐chain interactions without significantly perturbing the accessibility of the backbone NH‐groups at the interface of interacting proteins (Engen, 2003; Jensen & Rand, 2016). Noteworthy, both these conditions seem to apply to αT‐haemadin binding system. In fact, even though the *K*_d_ of full‐length for αT is in the sub‐picomolar range, the intrinsic affinity of free Haem(45–57) for exosite II is low (*K*_d_ ~ 13 μM) (Figure 7c), suggesting that even the C‐terminal tail in intact haemadin weakly interacts with exosite II, in keeping with the limited ionic strength dependence of haemadin binding to αT (Richardson et al., 2002) and with the weak affinity of the C‐terminal tail for exosite II, as estimated both experimentally and theoretically (see above). Furthermore, the x‐ray structure of αT‐haemadin complex shows that interactions at exosite II are loose and predominantly involve the long side chains of Arg‐ and Glu‐residues, with inter‐protein C^α^–C^α^ distances in the 12–18 Å range (1e0f.pdb) (Richardson et al., 2000). The results of [F]‐hirugen displacement and HDX‐MS experiments might be explained assuming the existence of an inter‐exosite negative allostery, as earlier proposed for αT (Chen et al., 2017; Petrera et al., 2009), whereby the binding of the 45–57 segment at exosite II would perturb long range exosite I and favor [F]‐hirugen release. However, this inter‐exosite allostery is expected to be bi‐directional (Verhamme et al., 2002), but nevertheless we did not observe any significant change in H/D exchange at exosite II after binding of hirugen or hirudin at exosite I (Figures S8 and S9). More likely, our results reflect a “direct” binding of the C‐terminal tail of haemadin, which has the N‐terminal domain still bound to the active site of the cognate αT molecule, to the exosite I of the protease in another αT‐haemadin molecular complex.

**SCHEME 1:**
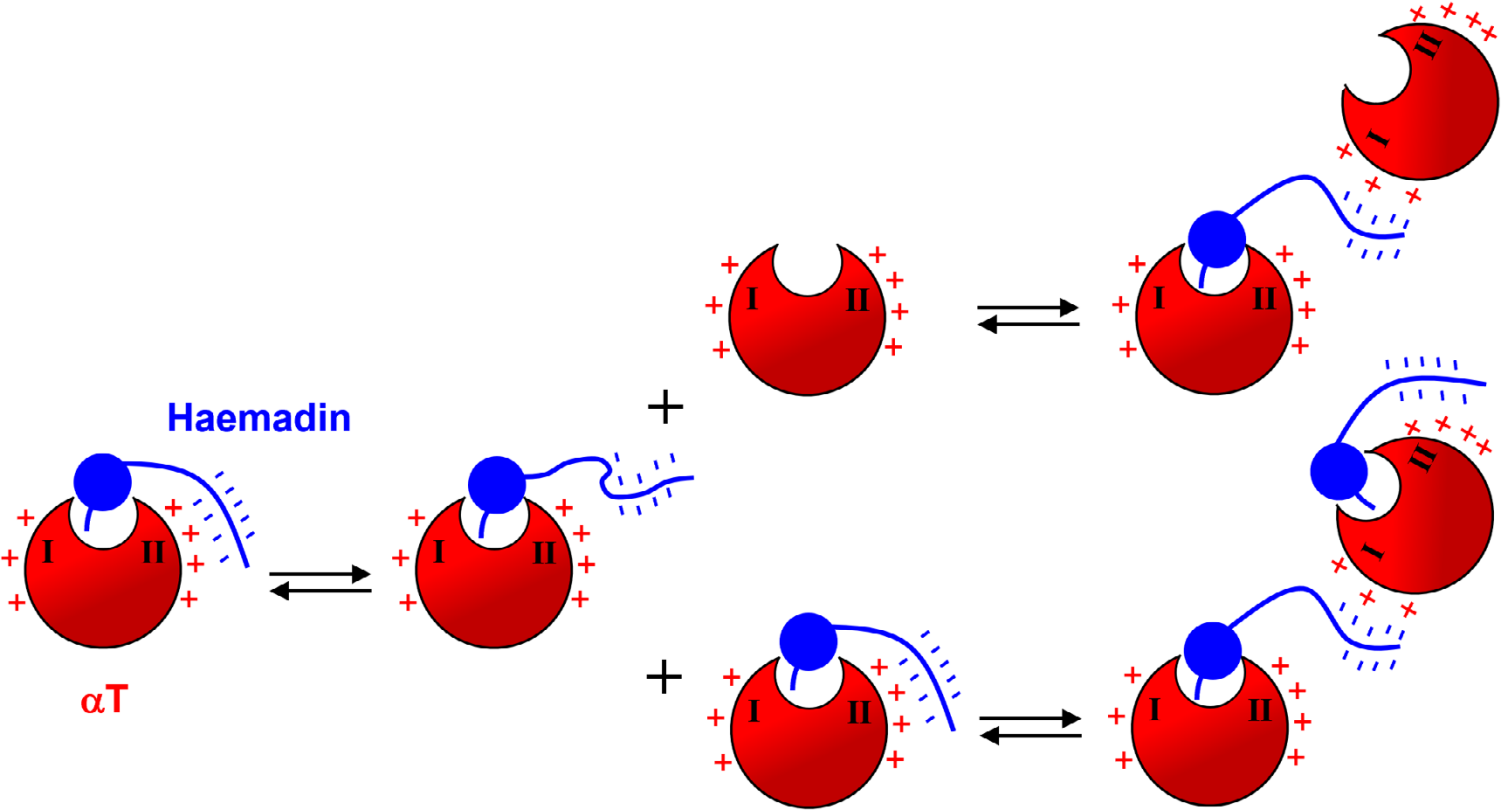
Proposed model explaining the promiscuity of haemadin binding to αT. At low αT concentrations, haemadin N‐terminal domain tightly interacts with the enzyme active‐site region and orients the negatively charged C‐terminal tail toward the highly electropositive exosite II. Despite the complementarity of electrostatic coupling, interactions of haemadin at exosite II are transient in nature, and therefore the C‐terminal tail exists in equilibrium between a bound and unbound form. At higher αT concentrations, such as those explored in this study in [F]‐hirugen displacement and HDX experiments or those that can be reached on the surface of activated platelets during the amplification phase of the coagulation cascade, the C‐terminal tail in its unbound form can interact with exosite I of another αT molecule nearby, isolated or in complex with haemadin.

The binding promiscuity of the 45–57 sequence, herein highlighted in the solution phase both for intact haemadin and the synthetic peptide Haem(45–57), is consistent with the picture emerging from the crystallographic structure of haemadin‐αT complex (Richardson et al., 2000) (see above) and can be explained considering that electrostatic interactions involving exosite II are transient/short lived in nature (Grassmann et al., 2023; Schreiber et al., 2009) and allow the haemadin C‐terminal tail in the protease‐inhibitor complex to “swing” between a αT‐bound and an unbound/free form. In the unbound form and at higher αT concentrations, the C‐terminal tail might interact with exosite I of another αT molecule nearby, either free or complexed to haemadin (see Scheme 1). It should be noted that the involvement of exosite I in αT binding to haemadin, highlighted by fluorescence displacement and HDX‐MS measurements, does not contradict the marginal effect that mutagenesis‐induced perturbation of exosite I has on the inhibitory properties of haemadin. In fact, enzyme inhibition assays were conducted at exceedingly low αT concentrations (i.e., 0.5 nM). Under these conditions, haemadin binding occurs in the active site region and at exosite II. However, at the much higher αT concentrations used in displacement (0.5 μM) and HDX (1.3 μM) experiments, the relative distance between αT molecules dramatically decreases and the C‐terminal tail (not permanently bound to the enzyme) might recognize exosite I of another αT molecule in its closer environment. These promiscuous interactions, occurring at relatively high αT concentrations, might also have a physio‐pathological relevance in vivo, during the amplification phase of the coagulation cascade, when there is a local burst of αT generation on the activated platelets surface.

### 3.3 Haemanorm as a novel bivalent, highly potent αT inhibitor

In this work, we have exploited the fragment‐based approach (Murray & Rees, 2009) to design and chemically synthesize haemanorm, a 29‐amino acid peptide in which the sequences 1–9 and 41–57 was joined through a 3xGly spacer to yield at the very first attempt haemanorm, a highly potent (*K*_I_ = 0.8 nM) and bivalent αT inhibitor. Indeed, enzyme inhibition assays indicate that haemanorm behaves as a tight binding competitive inhibitor of αT (Figure 8d). Furthermore, displacement experiments with PABA and [F]‐hirugen provide stringent (albeit indirect) evidence that haemanorm targets both the active site and exosite I (Figure 9a,b). A more direct and clear‐cut evidence for the bivalent nature of haemanorm binding to αT comes from HDX‐MS measurements showing well‐defined protection from H/D exchange of the enzyme active‐site, along with the regions contributing to the substrate specificity sites, and the loop‐regions specifying the exosite I (Figure 9c).

The fragment‐based approach is quite common in drug discovery and has been already exploited for the production of hirulogs, hirutonins, and hirunorms as simplified hirudin analogues (Lombardi et al., 1996; Maraganore et al., 1990; Szewczuk et al., 1993). Hirulogs and hirutonins, indeed, combine the exosite‐I binding properties of the C‐terminal hirudin sequence 48–65 with the ability of the tripeptide D‐Phe‐Pro‐Arg to moderately inhibit αT active site by forming an antiparallel short β‐sheet with Ser214‐Gly216 in a substrate‐like orientation. As a result, hirulog I (Angiomax®) is slowly cleaved at the Arg‐X bond, with a resulting less predictable pharmacological activity (Maraganore et al., 1990). To avoid inhibitor proteolysis by αT, hirunorms were developed after several runs of SAR studies. A suitable linker was identified that was able to orient the N‐terminal segment of hirudin (i.e., Ile‐Thr‐Tyr‐Thr‐Asp) into αT active site in a non‐substrate (hirudin‐like) fashion while allowing the C‐terminal hirudin sequence to interact with exosite I (Lombardi et al., 1996; Lombardi et al., 1999). As a result of these SAR studies, hirunorms are not cleaved by αT. In this work, we exploited the ability of haemadin N‐terminal segment (sequence 1–9) to bind αT active site in a non‐substrate mode and the intrinsic affinity of haemadin C‐terminal tail (sequence 41–57) for exosite I, to produce at the very first attempt a low‐molecular weight bivalent αT inhibitor (i.e., haemanorm) which is approximately 8‐ and 90‐fold more potent than hirulog I and hirunorm I, respectively, in inhibiting αT while its affinity is comparable with that of hirunorm IV and V, which are among the most potent reversible, bivalent αT inhibitors known so far (Lombardi et al., 1999).

## 4 CONCLUSIONS

Among natural protein anticoagulants isolated from blood‐feeding parasites, haemadin from *Haemadipsa sylvestris* has been proposed as promising alternative to hirudin and bivalirudin (Kostromina et al., 2021; Richardson et al., 2000; Strube et al., 1993). In fact, while haemadin retains the amidolytic inhibition of hirudin for αT, its unique binding to exosite II allows to inhibit not only free αT, but also the enzymatic activity of thrombomodulin‐bound αT on the vascular endothelial cell membranes. Conversely, hirudin impairs binding of all exosite‐I specific substrates, regardless of their pro‐coagulant (fibrinogen and PAR1) or anti‐coagulant (protein C zymogen) function. Furthermore, haemadin is not able to inhibit meizothrombin (Richardson et al., 2000), an important nicked/active intermediate of prothrombin along the pathway to αT generation, which activates Protein C 6‐fold more potently than αT (Stojanovski et al., 2018) and activates co‐factor V at least 50‐fold less efficiently than αT (Bradford & Krishnaswamy, 2019). At last, exosite‐I targeting by haemadin at relatively high αT concentrations, as highlighted in the study, might also interfere with fibrin generation by αT in the amplification phase of blood coagulation.

In the past, recombinant haemadin has been produced in the laboratory‐scale by means of periplasmic expression in *E. coli* as a fusion protein with maltose‐binding protein, followed by cleavage with factor Xa and ion exchange purification (Strube et al., 1993). Very recently, a semipreparative procedure, based on the self‐cleaving N‐ and C‐terminal intein fusion tag, followed by numerous chromatographic steps, has been developed for the production in *E. coli* of larger amounts of recombinant haemadin (Kostromina et al., 2021). The results reported in this work provide evidence that stepwise chemical synthesis, followed by in vitro disulfide oxidative refolding and RP‐HPLC purification, is a fast and versatile method to produce in good yields large amounts of fully active wild‐type haemadin preparations with pharmaceutical purity and to introduce both natural and non‐natural amino acids, suitable to improve the chemical stability of the protein inhibitor without appreciably losing activity. Although the unique features of αT inhibition by haemadin are attractive from a pharmaceutical point of view (see above), the real antithrombotic potential and safety profile of this inhibitor should be adequately evaluated in the course of in vivo studies using different models of thrombosis.

Another major achievement of this work entails the relatively facile production of haemanorm, a bivalent and non‐cleavable αT inhibitor targeting both the active and exosite I and displaying an inhibition potency higher than that of hirulog/bivalirudin (Angiomax®), already approved in the treatment of specific thrombotic diseases (Capranzano & Dangas, 2012). Haemanorm could represent a good starting point to design more potent hirulog‐like peptides or even “true” haemadin peptido‐mimetics recapitulating the binding specificity of the parent protein inhibitor.

## 5 MATERIALS AND METHODS

### 5.1 Reagents

Ecarin from *Echis carinatus* venom, human plasma fibrinogen, (D)‐Phe‐Pro‐Arg‐chloromethylketone (PPACK), and *p*‐aminobenzamidine (PABA) were purchased from Merck‐Sigma Aldrich (Darmstadt, Germany). The chromogenic substrate (D)‐Phe‐Pip‐Arg‐*p*‐nitroanilide (S2238) was from Chromogenix (Milan, Italy). N^α^‐Fmoc‐protected amino acids, solvents, and reagents for peptide synthesis were purchased from Applied Biosystems (Forster City, CA, USA) or Bachem AG (Bubendorf, Switzerland). Tris(2‐carboxyethyl)phosphine hydrochloride (TCEP‐HCl) and all other salts, solvents, and reagents were of analytical grade and purchased from Merck‐Sigma Aldrich (Darmstadt, Germany).

### 5.2 Chemical synthesis of haemadin

Wild type haemadin 1–57 and its Met5 → *nor*Leu analogue were synthesized by the solid‐phase method using the 9‐fluorenylmethyloxycarbonyl (Fmoc)/*t*‐butyl (*t*Bu) strategy (De Filippis et al., 1998) on a model PS3 automated synthesizer from Gyros Protein Technologies International (Tucson, AZ, USA). The peptide chain was assembled stepwise on a ChemMatrix resin (Matrix Innovation, Quebec, Canada) derivatized with Fmoc‐Lys (0.47 mmol/g). The removal of N^α^‐Fmoc‐protecting groups was achieved by treatment for 20 min with 20% piperidine in N‐methyl‐2‐pyrrolidone. Standard coupling reactions were performed with 2‐(1*H*‐benzotriazol‐1‐yl)‐1,1,3,3‐tetramethyluronium hexafluorophosphate (HBTU) and 1*H*‐hydroxy‐benzotriazole (HOBt) as activating agents, with a four‐fold molar excess of N^α^‐Fmoc‐protected amino acids (Novabiochem‐Sigma Aldrich, St. Louis, MO, USA) in the presence of diisopropylethylamine. For difficult couplings, involving Val‐, Ile‐, Leu‐, and Phe‐residues, a double coupling procedure was used along with the stronger activator 2‐(7‐aza‐1H‐benzotriazol‐1‐yl)‐1,1,3,3‐tetramethyluronium hexafluorophosphate (HATU) (Scapin et al., 2015). The following side‐chain protecting groups were used: *t*Bu for Asp, Glu Ser, Thr, and Tyr; *t*‐butyloxycarbonyl for Lys; triphenylmethyl for Cys, Asn and Gln; 2,2,4,6,7‐pentamethyldihydrobenzofuran‐5‐sulfonyl group for Arg. Stepwise chemical synthesis was interrupted at positions 26 and 8 and, before further proceeding, the corresponding intermediate synthetic peptides Cys26‐Lys57 and Val8‐Lys57 were analyzed by RP‐HPLC and high‐resolution mass spectrometry (HR‐MS) using a Waters (Milford, MO, USA) Xevo G2‐S Q‐TOF mass spectrometer. After peptide assembly was completed, the side chain‐protected peptidyl resin was treated for 120 min at room temperature with a mixture of trifluroacetic acid (TFA)/H_2_O/ethandithiol/triisopropylsilane (92:5:2:1 v/v/v/v). The resin was removed by filtration, and the acidic solution, containing the unprotected peptide, was precipitated with ice‐cold diethyl ether, and then lyophilized. The crude peptide with Cys‐residues in the reduced, free thiol form was fractionated by RP‐HPLC on a (4.6 × 250 mm, 5 μm granulometry, 300 Å pores size) C18 analytical column (Grace‐Vydac, Hesperia, CA), equilibrated with 0.1% (v/v) aqueous TFA and eluted with a linear acetonitrile‐0.078% (v/v) TFA gradient. The peptide material eluted in correspondence of the major chromatographic peaks was collected, lyophilized, and analyzed by HR‐MS, yielding mass values in agreement with the theoretical mass within 2 ppm accuracy (Table S1). Disulfide‐mediated oxidative renaturation of haemadin, to yield the correctly folded species, was carried out by dissolving the reduced purified polypeptide (0.4 mg/mL) in 0.1 M Tris–HCl pH 8.3 in the presence of 250 μM β‐ME for 24 h at 25 ± 0.1°C (Pozzi et al., 2010). The progression of the folding reaction was monitored by RP‐HPLC, using a (4.6 × 250 mm) C18 analytical column eluted with a linear acetonitrile‐0.078% (v/v) TFA gradient. For preparative purposes, aliquots (2 mg) of the refolding mixture, after 24‐h reaction time, were injected onto a Grace‐Vydac semi‐preparative (1.0 × 25 cm) C‐18 column eluted with a linear acetonitrile‐0.1% (v/v) TFA gradient from 15% to 45% in 30 min. The absorbance of the effluent was monitored at 220 nm. The material corresponding to the major peak was collected, lyophilized, analyzed by HR‐MS, and used for subsequent studies. All other peptides and the N^α^‐fluoresceinated analogues of hirugen and Haem(45–57) were synthesized, purified and characterized using the same strategy as reported above (Lancellotti et al., 2008; Li et al., 2010).

### 5.3 Production and expression of recombinant thrombin derivatives

The plasmid containing the cDNA of prethrombin‐2 (Pre2) was a generous gift from Prof. James A. Huntington (University of Cambridge, UK). Recombinant wild type and Pre2 mutants (i.e., S195A, F34A, R73A, R93A, R101A, K110A) were generated by site‐directed mutagenesis using primers purchased from Invitrogen (Carlsbad, CA). Recombinant Pre2 proteins were expressed, subjected to disulfide oxidative renatured, purified and activated to mature αT, as previously detailed (Li et al., 2010; Pontarollo et al., 2017; Pozzi et al., 2013). The chemical identity and purity of recombinant wild type and mutant thrombins was established by SDS‐PAGE (4%–12% acrylamide) (Figure S3) and HR‐MS (Table S1). Densitometric analysis of the electrophoretic gel bands was performed using the Geliance‐600 Chem‐Imaging system (Perkin‐Elmer, Waltham, MA, USA).

### 5.4 Spectroscopic measurements

The peptide/protein concentration was determined spectrophotometrically on a double‐beam V‐630 UV/Vis spectrophotometer (Jasco, Tokyo, Japan) using the molar absorptivity (*ε*, Μ^−1^∙cm^−1^) values reported in Table S1. For recombinant thrombins, a molar absorptivity of 66,390 M^−1^·cm^−1^ was used. For peptides lacking any suitable chromophore in the near‐UV region, the concentration was determined by weight.

CD spectra were recorded on a Jasco J‐810 spectropolarimeter equipped with a Peltier model ETC‐273 T temperature control system (De Filippis, Polverino de Laureto, et al., 1996). Far and Near UV‐CD spectra were recorded in a 0.1‐ or 1 cm‐pathlength quartz cell at a peptide/protein concentration of 0.47 or 1 mg/mL, respectively. All measurements were performed in phosphate buffered saline (PBS, 10 mM Na_2_HPO_4_ pH 7.4, 0.15 M NaCl) at 25 ± 0.1°C. The final spectra resulted from the average of four accumulations after base line subtraction. CD data were expressed as the mean residue ellipticity, [*θ*] = *θ*_obs_·MRW/(10·*l*·*c*), where *θ*_obs_ is the observed signal in degrees, MRW is the mean residue weight, *l* is the cuvette pathlength in cm, and *c* is the protein concentration in g∙mL^−1^. Stability measurements were carried out by recording the change of the CD signal in the far‐UV region as a function of sample temperature, using a 0.1‐cm pathlength cuvette and linear heating rate of 40°C∙h^−1^. The reversibility of thermal unfolding was determined by measuring the recovery of the CD signal upon cooling to the starting temperature. The melting temperature (*T*_m_) was estimated from the first‐derivative of the thermal denaturation curve (De Filippis, Vassiliev, et al., 1996; Frigerio et al., 1996).

Fluorescence measurements were performed at 25 ± 0.1°C in Tris buffered saline (TBS, 20 mM Tris–HCl, pH 7.4, 0.15 M NaCl, 0.1% PEG‐8000) in a 1 cm‐pathlength cuvette, using a Jasco FP‐6500 spectrofluorimeter, equipped with a Peltier model ETC‐273T temperature control system. Fluorescence binding measurements were recorded as previously reported (Acquasaliente et al., 2022; Pontarollo et al., 2017; Pozzi et al., 2016). Briefly, to a solution of αT (50 nM) were added, under gentle magnetic stirring, incremental volumes of ligand stock solutions. The samples were excited at 280 nm (5/5 excitation/emission slit) and the emission intensities were recorded at 334 nm, after subtracting the corresponding spectra of the ligands alone. Fluorescence data were corrected for sample dilution, which was always <2% at the end of the titration. To prevent possible inner filter effects, the optical density of the solution was kept lower than 0.05 AU at both *λ*_ex_ and *λ*_em_. A similar experimental setting was used in ligand displacement experiments with PABA and [F]‐hirugen (Pontarollo et al., 2017; Pozzi et al., 2016).

When the binding of PABA was studied, samples were excited at 336 nm and the emission of PABA was recorded at 376 nm, after baseline subtraction and correction for inner filter effect, as detailed elsewhere (Pontarollo et al., 2017). For the binding of [F]‐hirugen and [F]‐Haem(45–57), aliquots of αΤ S195A mutant stock solution were incrementally added to fluoresceinated peptide solutions. The samples were excited at 492 nm and the decrease in the fluorescence intensity of [F]‐peptide was recorded at 516 or 525 nm as a function of αT S195A concentration. According to the different binding systems under investigation, the equilibrium dissociation constants (*K*_D_) of αT‐ligand complexes were obtained by fitting the data points with Equation (1), describing a simple one‐site binding model:

(1) $$\frac{\left[\mathrm{RL}\right]}{\left[R\right]}=\frac{∆F}{∆F_{\max}}=\frac{\left[L\right]}{K_{d}+\left[L\right]},$$

or with Equation (2), describing a tight‐binding model:

(2) $$\begin{array}{rcl} \frac{\left[\mathrm{RL}\right]}{\left[R\right]} & = & \frac{∆F}{∆F_{\max}}\\ & = & \frac{\left(\left[R\right]+\left[L\right]+K_{d}\right)-\sqrt{{\left(\left[R\right]+\left[L\right]+K_{d}\right)}^{2}-4∙\left[R\right]∙\left[L\right]}}{2∙\left[R\right]}, \end{array}$$

where [*R*] and [*L*] are the αT or ligand concentrations, and [*RL*] is the complex concentration. The interpolation of the data points was performed using the computer program Origin vs. 2019b (Microcal, CA, USA).

### 5.5 Enzyme inhibition assays and data analysis

The concentration of natural and recombinant thrombins was determined by active‐site titration with PPACK in the presence of S2238 as a chromogenic substrate, using the procedure reported elsewhere (Pozzi et al., 2016), and was found to be identical (within 5% error) to that determined spectrophotometrically. Enzyme inhibition assays were all performed at 25 ± 0.1°C in TBS (10 mM Tris–HCl pH 7.4, 0.1% PEG‐8000 w/v) in the presence of 0.15 M or 0.4 M NaCl, using a Jasco double‐beam V‐630 spectrophotometer equipped with a Peltier model PAC‐740 temperature control system (De Filippis et al., 1999). Tight‐binding inhibition assays were performed by pre‐incubating αT (500 pM) for 30 min with increasing inhibitor concentrations [I]. The reaction started by the addition of the chromogenic substrate S2238 (20 μM) and the release of *p‐*nitroaniline (*p*NA) was continuously monitored at 405 nm, using a molar absorptivity of 9920 M^−1^∙cm^−1^. The apparent inhibition constant, *K*_I_^app^, was determined as a fitting parameter by interpolating the data points to Equation (3):

(3) $$\frac{v_{i}}{v_{o}}=1-\frac{\left(\left[E\right]+\left[I\right]+K_{I}^{\mathrm{app}}\right)-\sqrt{{\left(\left[I\right]+\left[E\right]+K_{I}^{\mathrm{app}}\right)}^{2}-4∙K_{I}^{\mathrm{app}}∙\left[E\right]}}{2∙\left[E\right]},$$

where [*E*] is the total αT concentration, while *v*_0_ and *v*_i_ are the steady state velocities of S2238 hydrolysis in the absence or presence of *I*. Under the assumption of competitive inhibition, *K*_I_^app^ can be converted into the real inhibition constant, *K*_I_, using Equation (4):

(4) $$K_{I}=\frac{K_{I}^{\mathrm{app}}}{1+\frac{\left[S\right]}{K_{m}}},$$

where [*S*] is the substrate concentration (40 μM) and *K*_m_ is the Michaelis constant of S2238 hydrolysis by αT at 25°C (*K*_m_ = 7 μM).

When slow binding inhibition assays were performed, αT (50 pM) was added to solutions of S2238 (50 μM) in the presence of increasing inhibitor (*I*) concentrations. From the time‐course release of pNA, [*P*]_t_, an observed kinetic constant (*k*_obs_) of substrate hydrolysis could be determined as a fitting parameter by interpolating each progress curve at increasing [*I*] to Equation (5) (Arcone et al., 2009):

(5) $${\left[P\right]}_{t}=v_{s}∙t+\frac{v_{o}-v_{s}}{k_{\mathrm{obs}}}∙\left(1-e^{-k_{\mathrm{obs}}∙t}\right),$$

where *v*_0_ and *v*_s_ is the initial or steady state velocity at each [*I*]. The association (*k*_on_) and dissociation (*k*_off_) rate constants of αT‐haemedin interaction were determined as fitting parameters by interpolating the data points of *k*_obs_ as a function of [*I*] with Equation (6):

(6) $$k_{\mathrm{obs}}=\frac{k_{\mathrm{on}}}{1+\frac{\left[S\right]}{K_{m}}}∙\left[I\right]+k_{\mathrm{off}}.$$

From the values of *k*_on_ and *k*_off_, the equilibrium dissociation constant of the αT‐haemadin complex was obtained as *K*_I_ = *k*_off_/*k*_on_.

### 5.6 Hydrogen–deuterium exchange mass spectrometry

HDX‐MS measurements were performed using a Xevo G2S Q‐TOF (Waters, Milford, MO, USA) mass spectrometer, equipped with a standard electrospray ionization source, an Acquity M‐class UPLC (Waters), an Automation 2.0 sample workstation (Waters), and an HDX PAL autosampler (Leap technologies, Carrboro, NC, USA), as previously reported (Peterle et al., 2020). Leu‐enkephalin (Waters) was continuously infused as the reference lock mass. αT (30 μM) was incubated with saturating concentrations of haemadin or inhibitor/ligand peptides in H_2_O‐PBS for 1 h at 20°C. Thereafter, at each time point (15 s, 1 min, 5 min, 30 min), an aliquot (3 μL) of the αT‐peptide complex solution was diluted 20‐fold in deuterated buffer (i.e., 20 mM sodium phosphate in 95:5 D_2_O:H_2_O solution, pD 7.4, containing 150 mM NaCl) to have a αT saturation of at least 85%. For each sample, H/D exchange was quenched at 0°C by addition of an equal volume of quenching buffer, that is, 20 mM sodium phosphate in H_2_O, containing 0.5 M TCEP‐HCl and 3 M guanidinium hydrochloride, adjusted to pH 2.35. The pH and pD values were measured at 25°C, using a Mettler‐Toledo (Columbus, OH, USA) mod. Fiveasy Plus pH‐meter. Quenched samples were immediately injected onto an Enzymate™ BEH Pepsin Column (Waters), thermostated at 15°C and eluted at a constant flow rate (100 μL/min) with 0.23% (v/v) aqueous formic acid. The resulting peptic fragments were online trapped on an Acquity UPLC BEH C18 VanGuard Pre‐column (Waters), and eluted on an Acquity UPLC BEH C18 column (Waters) with a linear acetonitrile‐0.1% formic acid gradient from 5% to 60% in 10 min, at a constant flow rate of 40 μL/min. The effluent was analyzed online with the Xevo G2S Q‐TOF spectrometer in the resolution mode (m/z 50–2000) and each peptic fragment was identified in the MS^E^ mode, using argon as collision gas. Unlabeled proteins were prepared as control samples in the same manner as those that were labeled with deuterium. Fragments generated from online pepsin digestion of αT were identified using Protein Lynx Global Server 3.0 (Waters) and then analyzed with DynamX 3.0 software (Waters). The average sequence coverage in HDX experiments was 89.5%, with a redundancy (i.e., the average number of overlapping peptic fragments for each amino acid) between 2.5 and 4.0. Only those peptic fragments that met the following criteria were considered for the HDX analysis: (i) 5% retention time window in chromatographic separation; (ii) maximum MH^+^ error of 6 ppm; (iii) at least 2 ion products identified for each peptic fragment; (iv) *R* value >0.2 for each fragment, where *R* is the ratio of the number of product ions per amino acid residue; (v) fragments containing less than 4 or more than 33 amino acids were not considered, due to increased ambiguity and limited sequence localization. Because only the relative levels of deuterium uptake of individual fragments were compared, no back‐exchange correction was performed. In fact, it is not necessary to correct for back‐exchange when the same protein is studied in two or more states (e.g., bound vs. unbound) and deuteration levels are compared; when the generated peptides will be the same for both states and the experimental variables in the LC–MS instrumentation remain constant, all samples experience the same level of back‐exchange in the post‐quench steps (Masson et al., 2019; Peterle et al., 2022; Wales & Engen, 2006). In this study, we used the same αT preparation and the samples were subjected to the same procedure for exchange, quenching, digestion and analysis. Therefore, back‐exchange can be safely considered to be constant between different ligand binding experiments and to not affect data interpretation (see Figure S11).

All HDX measurements were carried out in triplicate, with the error as standard deviation. ANOVA analysis and t tests with a p‐value cut‐off of 0.05, as implemented in the DECA program (github.com/komiveslab/DECA) (Peacock & Komives, 2021), were used to determine the significance of differences between HDX data points.

### 5.7 Computational methods

Modeling studies were performed using the Molecular Operating Environment (MOE) vs. 15 software and the OPLS‐AA force field (Chemical Computing Group, Montreal, QC, Canada). Electrostatic potential calculations were performed using the APBS software (Baker et al., 2001). For αT, calculations were run on the non‐glycosylated x‐ray structure of αT (1 ppb), after removal of the coordinates of the inhibitor D‐Phe‐Pip‐Arg‐chloromethylketone (PPACK), water, and HEPES molecules (Bode et al., 1992). The electrostatic contribution of the Na^+^‐ion bound to αT was not considered in our calculations. A solvent dielectric constant of 78.14 and a protein dielectric constant of 2.0 at 310 K in 150 mM NaCl were used. The final electrostatic maps were constructed by subtracting the protein self‐energies from the calculated map using the DXMATH utility in APBS. Protein structure images were generated using the PyMOL vs. 1.3 Molecular Graphics System (DeLano Scientific, San Diego, CA.

## AUTHOR CONTRIBUTIONS

Laura Acquasaliente and Nicola Pozzi performed the biochemical work; Andrea Pierangelini and Anna Pagotto performed HDX‐MS analyses; Laura Acquasaliente, Nicola Pozzi, and Vincenzo De Filippis designed research; Laura Acquasaliente wrote a very preliminary draft of the manuscript; Vincenzo De Filippis conceived and coordinated the work and wrote the final version of the manuscript; all authors analyzed and interpreted the data and approved the final content of the manuscript.

## FUNDING INFORMATION

This work was supported by a Grant from the CaRiPaRo Foundation Excellence Research Project—BPiTA n. 52012 to V.D.F. and by PRID‐Junior 2019 Project to L.A.

## CONFLICT OF INTEREST STATEMENT

The authors declare no conflicts of interest.

## Supporting information


**Data S1.** Supplementary materials.

## Data Availability

All other data that support the findings of this study are available from the corresponding author upon reasonable request.
