# Supplementary material for: From haemadin to haemanorm: Synthesis and characterization of full‐length haemadin from the leech Haemadipsa sylvestris and of a novel bivalent, highly potent thrombin inhibitor (haemanorm)
Source: Protein Sci. 2023 Dec 1;32(12):e4825. doi: 10.1002/pro.4825 (PMC10683372; doi:10.1002/pro.4825)
Supplement: Supplementary file 1 — Data S1. Supplementary materials. [file PRO-32-e4825-s001.pdf]

Supplementary Material

From Haemadin to Haemanorm: Synthesis and Characterization of Full-length Haemadin from the leech *Haemadipsa sylvestris* and of a Novel Bivalent, Highly Potent Thrombin Inhibitor (Haemanorm)

Laura Acquasaliente [1,^], Andrea Pierangelini [1,^], Anna Pagotto [1], Nicola Pozzi [1,#] and Vincenzo De Filippis [1,*]

[1] Laboratory of Protein Chemistry & Molecular Haematology, Department of Pharmaceutical and Pharmacological Sciences, School of Medicine, University of Padova.

[#] **Current address**: Department of Biochemistry and Molecular Biology, Edward A. Doisy Research Center, Saint Louis University

*** To whom correspondence should be addressed**: Prof. Vincenzo De Filippis, Department of Pharmaceutical and Pharmacological Sciences, School of Medicine, University of Padua, via Marzolo 5, Padua, 35131 Italy. Phone: (+39) 0498275698, e-mail: vincenzo.defilippis@unipd.it

^ These authors equally contributed to this work

Running title: From Haemadin to Haemanorm

Table S1. Mass spectrometry data and molar absorptivity values of the synthetic peptides produced in this study.

**Protein/Peptide**	**MW (experimental) [a] a.m.u**	**MW (theoretical) a.m.u.**	**Molar absorptivity (ε) $M^{-1} \cdot cm^{-1}$**
Haemadin(1-57)	6251.98 ± 0.41	6251.90	ε^{280nm} 3355
Haem(45-57)	1659.54 ± 0.13	1659.68	ε^{257nm} 400
[F]-Haem(45-57)	2017.99 ± 0.24	2018.02	ε^{492nm} 68000
Haem(1-10)	1061.32 ± 0.11	1061.30	ε^{257nm} 200
ψHaem(1-10) Met5Nle	1042.66 ± 0.24	1042.64	ε^{257nm} 200
ψHaem(1-10) Phe3Tyr	1058.77 ± 0.18	1058.66	ε^{280nm} 1490
ψHaem(1-10) Phe3Trp	1081.61 ± 0.22	1081.64	ε^{280nm} 5580
ψHaem(1-10) Phe3Bip	1118.66 ± 0.15	1118.60	ε^{280nm} 4266
ψHaem (1-10) Phe3DiP	1118.74 ± 0.19	1118.68	ε^{257nm} 200
ψHaem(1-10) Phe3αNal	1092.66 ± 0.31	1092.60	ε^{280nm} 6200
ψHaem(1-10) Phe3βNal	1092.46 ± 0.33	1093.35	ε^{280nm} 5380
Haemanorm	3258.55 ± 0.42	3258.60	ε^{257nm} 600
Hirugen(54-65)	1468.50 ± 0.11	1468.55	ε^{280nm} 1280
[F]-Hirugen(54-65)	1858.29 ± 0.16	1858.20	ε^{492nm} 68000

[a] The values reported refer to the average masses.

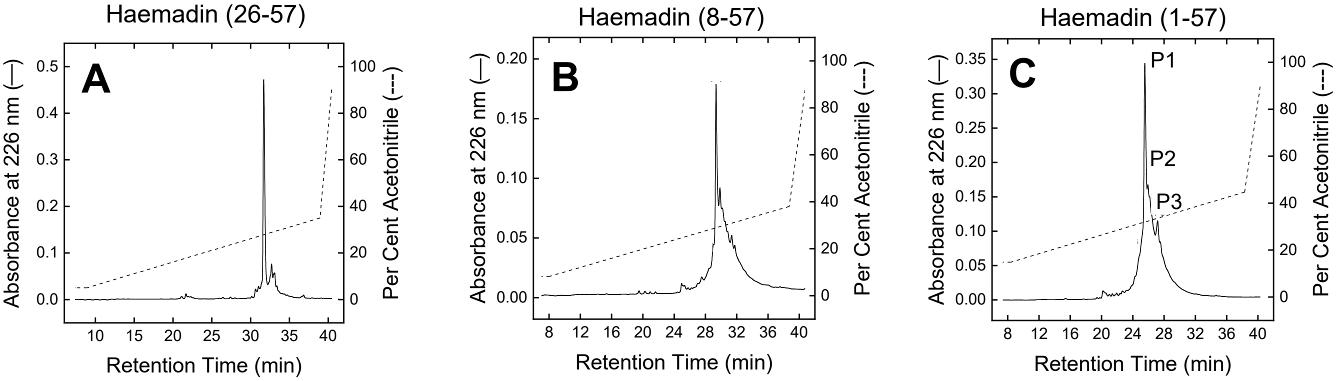

Figure S1. Chemical synthesis and characterization of intermediate peptides during the stepwise synthesis of Haemadin. RP-HPLC analysis of the synthesis reaction mixtures of Haem(26-57) (**A**), Haem(8-57) (**B**) and full-length Haemadin (1-57) (**C**), after resin cleavage and side-chain deprotection. Aliquots (10 μg) of each intermediate peptides were injected onto a Zorbax C18 analytical column (4.6 mm x 150 mm), eluted with a linear acetonitrile gradient (---) in 0.1% (v/v) aqueous TFA at a flow rate of 0.8 ml/min. The peptide materials eluted in correspondence of the major chromatographic peaks were collected and analysed by high-resolution MS (see **Supplementary Table S1**). RP-HPLC analysis of the crude peptide, with Cys-residues in the reduced form (R-Haem) shows a major peak (P1) and two minor components (P2 and P3) eluting as shoulders at slightly higher retention times (r.t.) (Supplementary Figure S1). Whereas the material eluting with P3 has a mass value (6313.76 a.m.u.) compatible with the presence of a residual tBu protecting group, remaining after resin cleavage, P1 and P2 display identical mass values (average mass: 6257.74 ± 0.02 a.m.u.), matching the average theoretical mass of R-haem (6257.80 a.m.u.), while the slight difference in r.t. might be caused by different cis-trans conformers of one (or more) of the four Pro-residues present in the haemadin polypeptide chain (**Figure 2A**), resulting in different interaction with the RP stationary phase and different r.t. values [O'Neal KD, Chari MV, McDonald CH, Cook RG, Yu-Lee LY, Morrisett JD, Shearer WT. 1996. Multiple cis-trans conformers of the prolactin receptor proline-rich motif (prm) peptide detected by reverse-phase hplc, cd and nmr spectroscopy. Biochem J. 315:833-844].

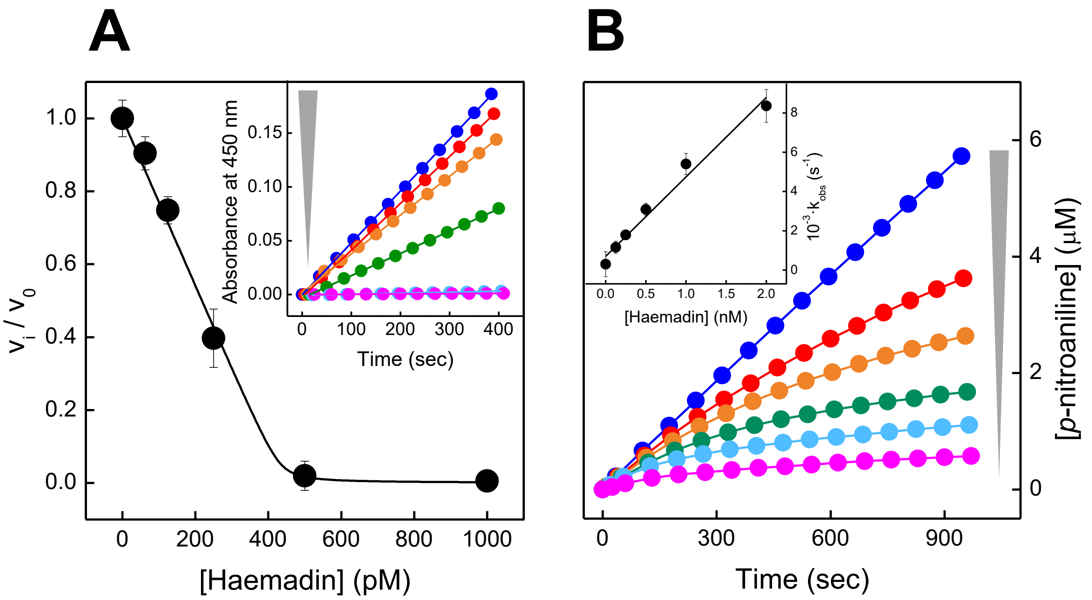

Figure S2. Inhibition of thrombin amidolytic activity by Haemadin. (**A**) Tight-binding inhibition. αT solutions (500 pM) were incubated with increasing concentrations of haemadin and added with the chromogenic substrate S2238 (100 μM). The values of v_i/v_0, were plotted as a function of the inhibitor, as indicated, where v_0 and v_i are the steady state velocities in the absence or presence of the inhibitor. The data points were interpolated with eq. 3, to yield K_i^{app} , which was then converted to K_I using eq. 4. **(B)** Slow-binding inhibition. Assays were performed by adding αT (50 pM) into a solution of S2238 (40 μM) in the presence of increasing concentrations of haemadin, as indicated. Each progress curve was interpolated with eq. 5 to obtain the corresponding observed kinetic constant, k_{obs}. Interpolation of the data points with eq. 6 in the plot of k_{obs} vs. [I], allowed us to estimate the values of k_{on} and k_{off} (see Methods for details). All measurements were conducted in at 25 ± 0.1°C in TBS, pH 7.4. The data points are the average of three independent measurements, with error bars as ±SD.

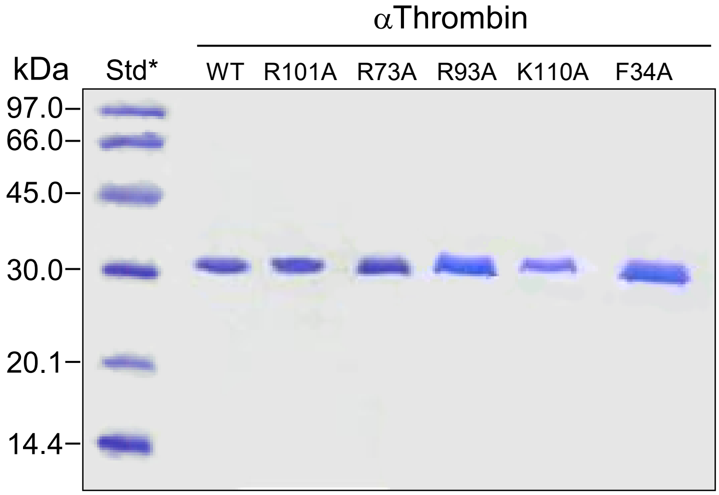

Figure S3. Electrophoretic analysis of purified recombinant αT mutants. An aliquot (5 μg) of purified mutant protein solutions was analysed by SDS-PAGE (4 - 12% acrylamide) under reducing conditions and Coomassie stained. Densitometric analysis of the gel bands allowed to estimate a purity > 98% for the recombinant wild type and mutant proteins. The gel bands correspond to thrombin B-chains (~31 kDa), whereas the A-chains (~2 kDa) are not visible. *Std: molecular weight protein standard.

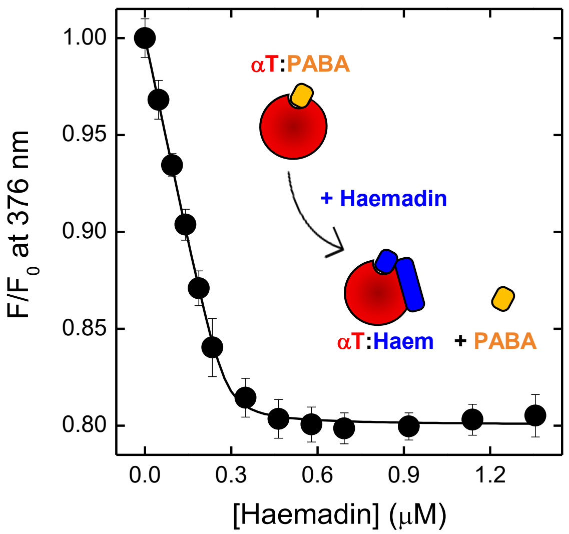

Figure S4. Displacement of PABA from the primary specificity S1 site of αT by haemadin. A solution of αT (60 nM) in TBS, pH 7.4, was first incubated at 25 ± 0.1 °C with a saturating concentration of PABA (500 nM) and then added with increasing haemadin concentrations. Samples were excited at 335 nm and the release of PABA was monitored by recording the decrease of fluorescence signal at 376 nm, after base line subtraction. The data are reported as F/F_0 ratio, where F_0 and F are the emission intensities of PABA in the absence and presence of haemadin. The data points are the average of three independent measurements, with errors as ± SD.

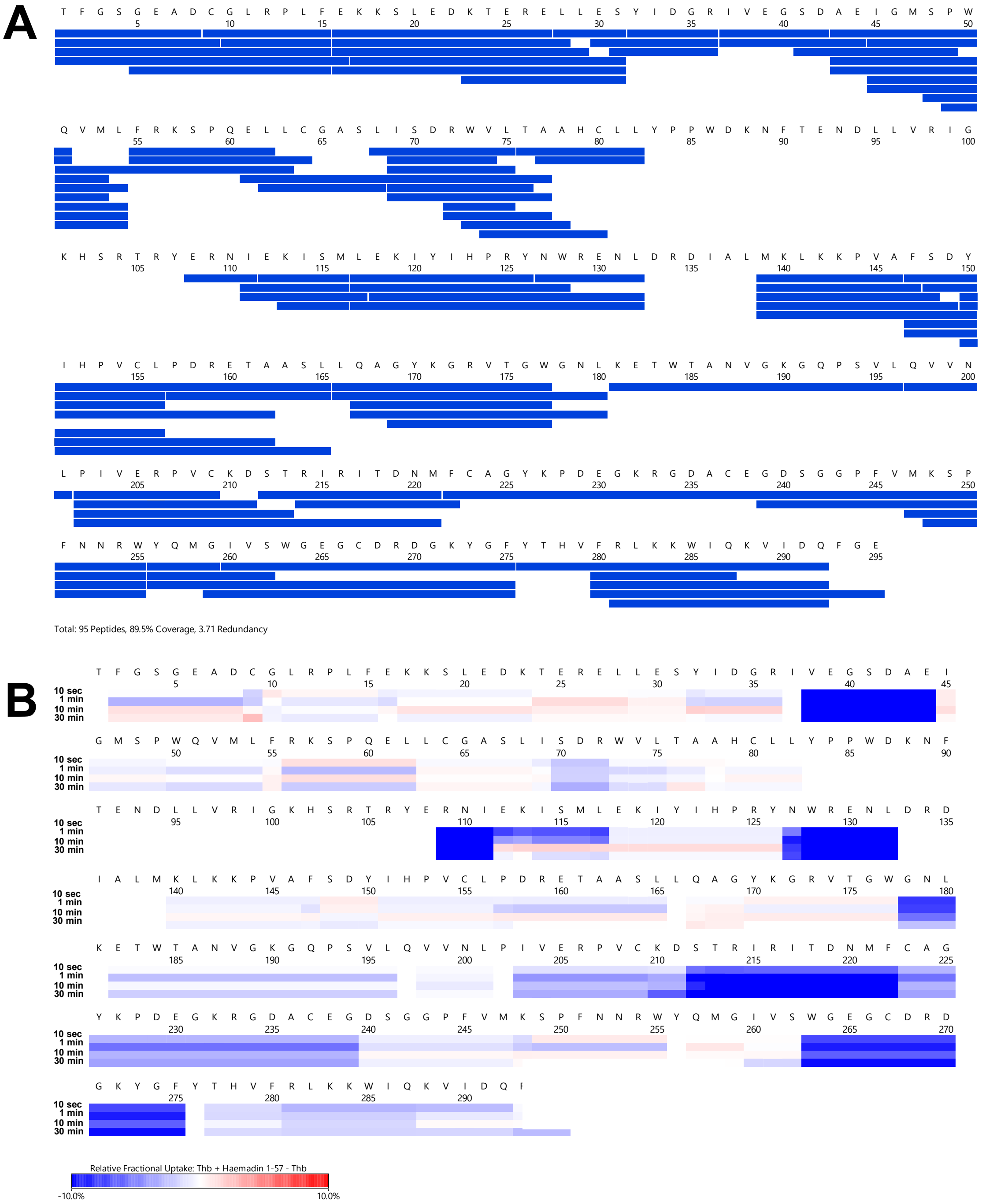

Figure S5. (**A**) Sequence coverage of αT after peptide mass fingerprint analysis with pepsin. Blue bars represent the peptic fragments that were identified, according to the analytical criteria reported in the Methods. (**B**) Difference bi-dimensional heatmap (%ΔD) of the per cent deuterium uptake (%D) of αT with and without Haemadin, at increasing incubation times (15 sec – 30 min). %D data of αT alone were subtracted from the corresponding data obtained in the presence of saturating Haemadin concentration. The color key in the heatmap indicates %ΔD, from dark blue (at least -10% deuterium uptake) to light red, where negative %D differences indicate that those region in αT become more protected from H/D exchange in the presence of Haemadin, compared to free αT. Only those regions displaying %ΔD > ± 4% were considered significant and used for building the three-dimensional heatmap reported in **Figure 5** of the main text. The heatmap was generated using 95 single and overlapping fragments. αT numbering used by the Dynamix software in HDX analysis is different from the classical chymotrypsin numbering (used for building the three-dimensional heatmap in **Figure 5**) and goes from Thr1 of the A-chain to Glu295 of the B-chain.

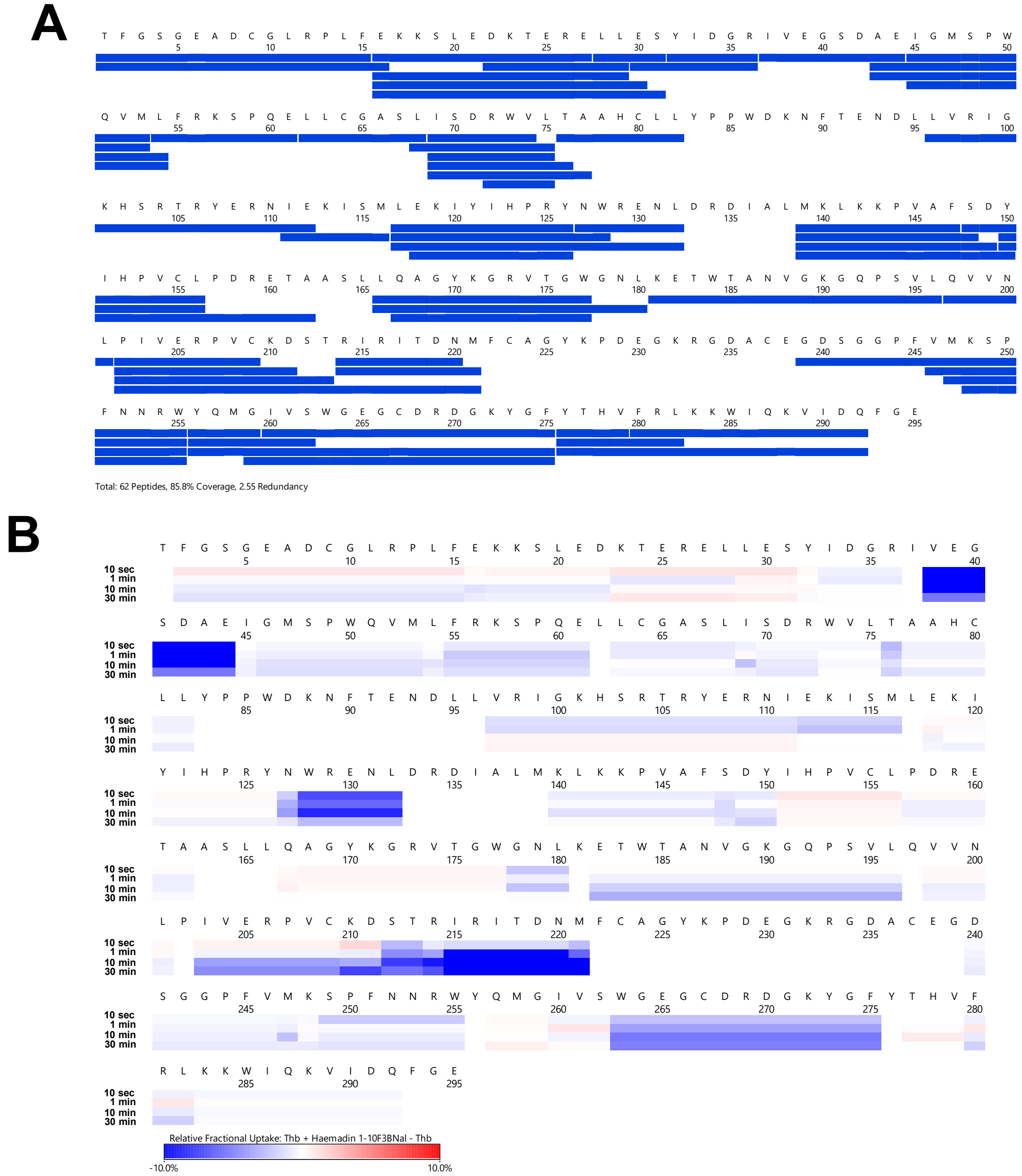

Figure S6. **HDX-MS analysis of ψHaem(1-10)Phe3βNal binding to αT.** (**A**) Sequence coverage of αT after peptide mass fingerprint analysis with pepsin. Blue bars represent the peptic fragments that were identified, according to the analytical criteria reported in the **Methods**. (**B**) Difference bi-dimensional heatmap (%ΔD) of the per cent deuterium uptake (%D) of αT with and without ψHaem(1-10)Phe3βNal peptide, at increasing incubation times (15 sec – 30 min). %D data of αT alone were subtracted from the corresponding data obtained in the presence of saturating peptide concentration. The color key in the heatmap indicates %ΔD, from dark blue (at least -10% deuterium uptake) to light red, where negative %D differences indicate that those region in αT become more protected from H/D exchange in the presence of the inhibitor, compared to αT alone. Only those regions displaying %ΔD > ± 4% were considered significant and used for building the three-dimensional heatmap reported in **Figure 6** of the main text. The heatmap was generated using 62 single and overlapping fragments. αT numbering used by the Dynamix software in HDX analysis is different from the classical chymotrypsin numbering (used for building the three-dimensional heatmap in **Figure 6**) and goes from Thr1 of the A-chain to Glu295 of the B-chain.

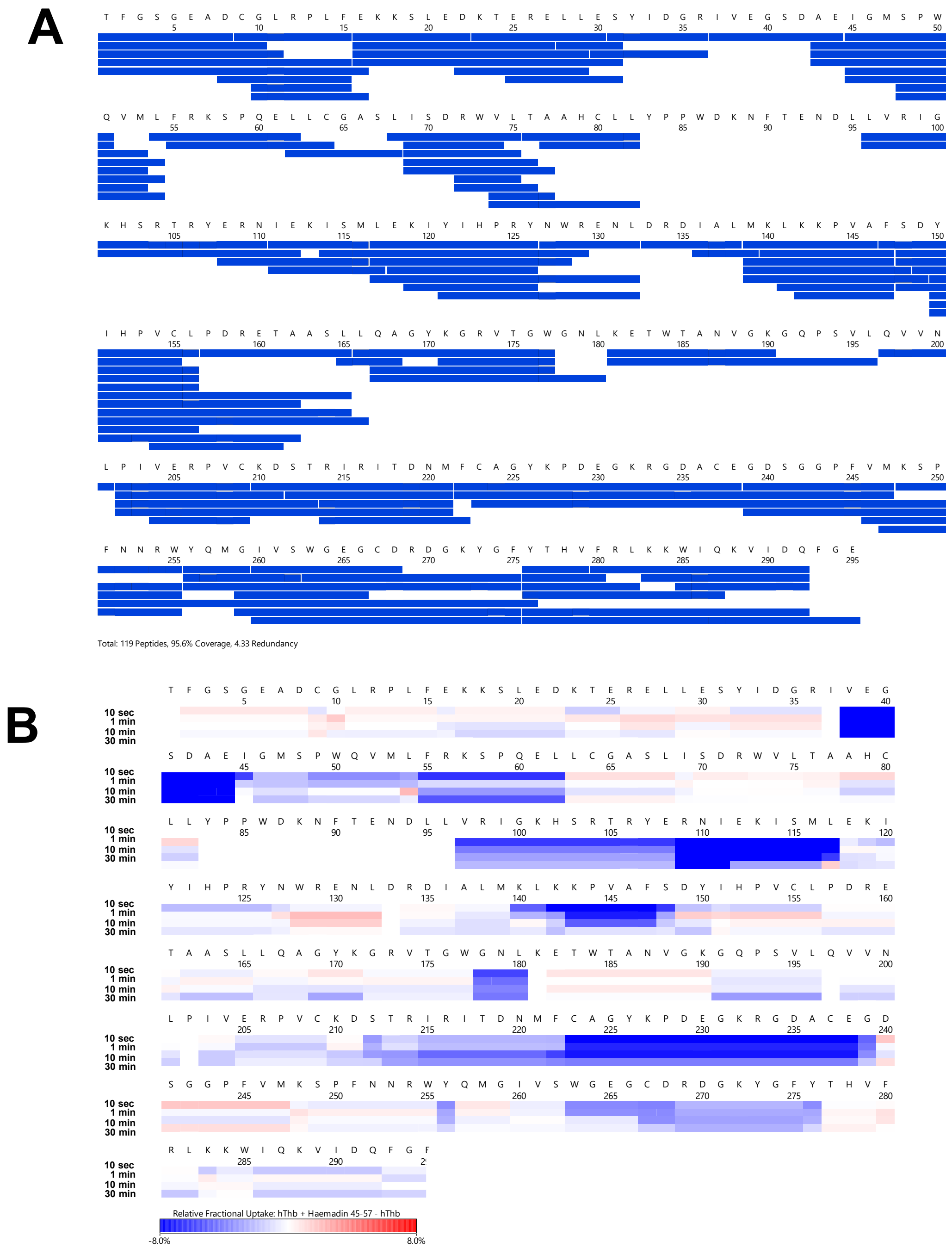

Figure S7. **HDX-MS analysis of Haem(45-57) binding to αT.** (**A**) Sequence coverage of αT after peptide mass fingerprint analysis with pepsin. Blue bars represent the peptic fragments that were identified, according to the analytical criteria reported in the **Methods**. (**B**) Difference bi-dimensional heatmap (%ΔD) of the per cent deuterium uptake (%D) of αT with and without Haem(45-57) peptide, at increasing incubation times (15 sec – 30 min). %D data of αT alone were subtracted from the corresponding data obtained in the presence of saturating peptide concentration. The color key in the heatmap indicates %ΔD, from dark blue (at least -10% deuterium uptake) to light red, where negative %D differences indicate that those region in αT become more protected from H/D exchange in the presence of the ligand peptide, compared to αT alone. Only those regions displaying %ΔD > ± 4% were considered significant and used for building the three-dimensional heatmap reported in **Figure 7** of the main text. The heatmap was generated using 119 single and overlapping fragments. αT numbering used by the Dynamix software in HDX analysis is different from the classical chymotrypsin numbering (used for building the three-dimensional heatmap in **Figure 7**) and goes from Thr1 of the A-chain to Glu295 of the B-chain.

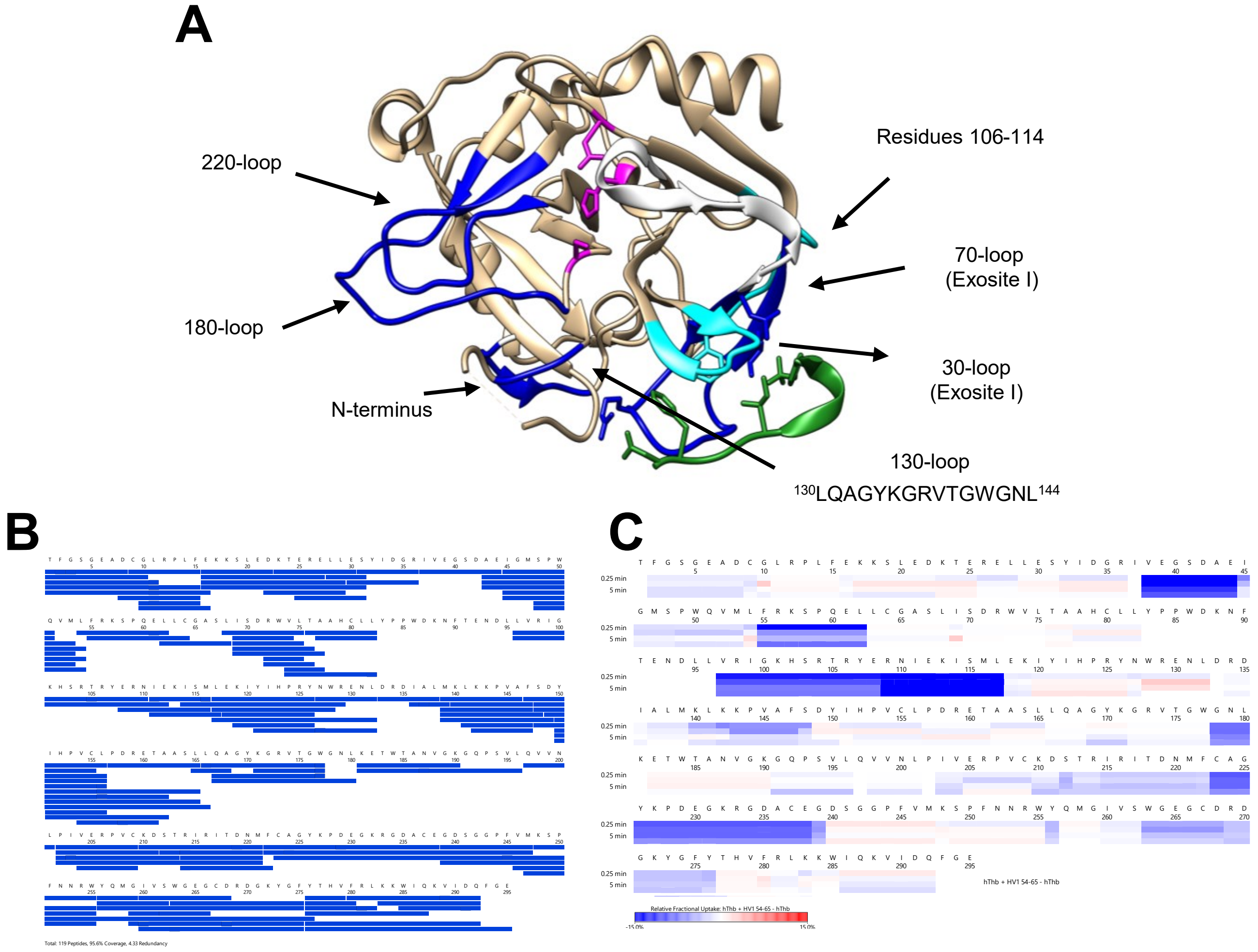

Figure S8. **HDX-MS analysis of Hirugen binding to αT**. (**A**) Three-dimensional HDX-MS difference map of deuterium uptake by αT in the absence and presence of Hirugen (green). The values of deuterium uptake were mapped onto the crystal structure of αT-Hirugen complex (2jho.pdb). The regions that are protected from H/D exchange at shorter incubation times (15 – 60 sec) are coloured in cyan, whereas the region that show protection even at longer incubation times (30 min) are in dark blue; the regions that displayed any change in HDX are coloured in light orange, while the regions that were not covered in the peptic map are in white. The catalytic amino acids are shown in magenta. Active-site amino acids (H57, D102, S195) are shown in magenta. Experimental conditions were as follows: 20 °C in 20 mM sodium phosphate in 95:5 D_2O:H_2O solution, pD 7.4, containing 150 mM NaCl, at a αT-Hirugen complex concentration of 1.35 μM. HDX-MS measurements were conducted in three independent measurements. (**B**) Sequence coverage of αT after peptide mass fingerprint analysis with pepsin. Blue bars represent the peptic fragments that were identified, according to the analytical criteria reported in the **Methods**. (**C**) Difference bi-dimensional heatmap (%ΔD) of the per cent deuterium uptake (%D) of αT with and without Haemanorm, at increasing incubation times (15 sec – 30 min). %D data of αT alone were subtracted from the corresponding data obtained in the presence of saturating inhibitor concentration. The color key in the heatmap indicates %ΔD, from dark blue (at least -10% deuterium uptake) to light red, where negative %D differences indicate that those region in αT become more protected from H/D exchange in the presence of the ligand peptide, compared to αT alone. Only those regions displaying %ΔD > ± 4% were considered significant and used for building the three-dimensional heatmap reported in **panel A**. The heatmap was generated using 119 single and overlapping fragments. αT numbering used by the Dynamix software in HDX analysis is different from the classical chymotrypsin numbering (used for building the three-dimensional heatmap in **panel A**) and goes from Thr1 of the A-chain to Glu295 of the B-chain.

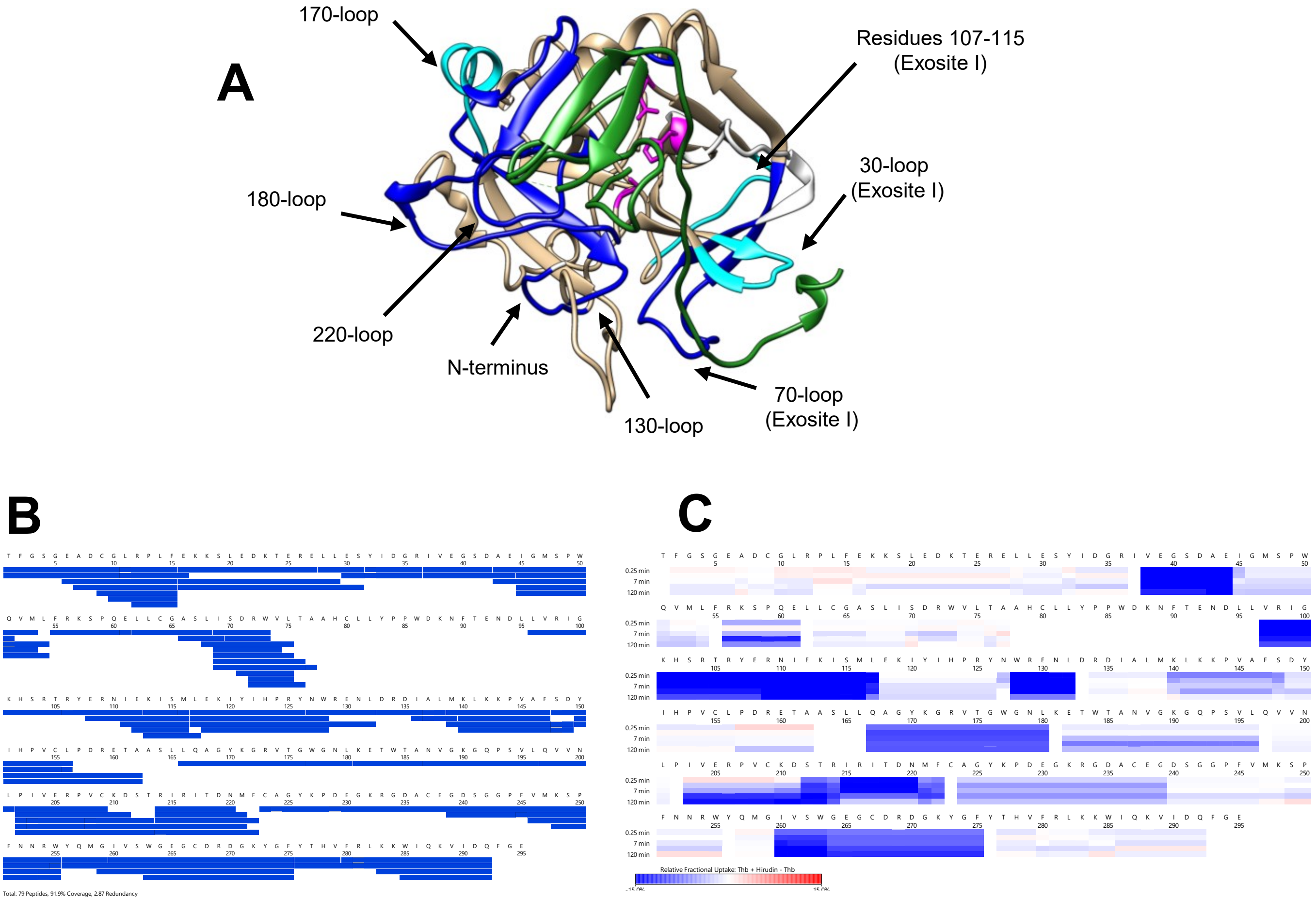

Figure S9. **HDX-MS analysis of Hirudin binding to αT**. (**A**) Three-dimensional HDX-MS difference map of deuterium uptake by αT in the absence and presence of Hirugen (green). The values of deuterium uptake were mapped onto the crystal structure of αT-Hirugen complex (4htc.pdb). The regions that are protected from H/D exchange at shorter incubation times (15 – 60 sec) are coloured in cyan, whereas the region that show protection even at longer incubation times (30 min) are in dark blue; the regions that displayed any change in HDX are coloured in light orange, while the regions that were not covered in the peptic map are in white. The catalytic amino acids are shown in magenta. Active-site amino acids (H57, D102, S195) are shown in magenta. Experimental conditions were as follows: 20 °C in 20 mM sodium phosphate in 95:5 D_2O:H_2O solution, pD 7.4, containing 150 mM NaCl, at a αT-Hirudin complex concentration of 1.35 μM. HDX-MS measurements were conducted in three independent measurements. (**B**) Sequence coverage of αT after peptide mass fingerprint analysis with pepsin. Blue bars represent the peptic fragments that were identified, according to the analytical criteria reported in the Methods. (**C**) Difference bi-dimensional heatmap (%ΔD) of the per cent deuterium uptake (%D) of αT with and without Hirudin, at increasing incubation times (15 sec – 30 min). %D data of αT alone were subtracted from the corresponding data obtained in the presence of saturating inhibitor concentration. The color key in the heatmap indicates %ΔD, from dark blue (at least -10% deuterium uptake) to light red, where negative %D differences indicate that those region in αT become more protected from H/D exchange in the presence of Hirudin, compared to αT alone. Only those regions displaying %ΔD > ± 4% were considered significant and used for building the three-dimensional heatmap reported in **panel A**. The heatmap was generated using 79 single and overlapping fragments. αT numbering used by the Dynamix software in HDX analysis is different from the classical chymotrypsin numbering (used for building the three-dimensional heatmap in **panel A**) and goes from Thr1 of the A-chain to Glu295 of the B-chain.

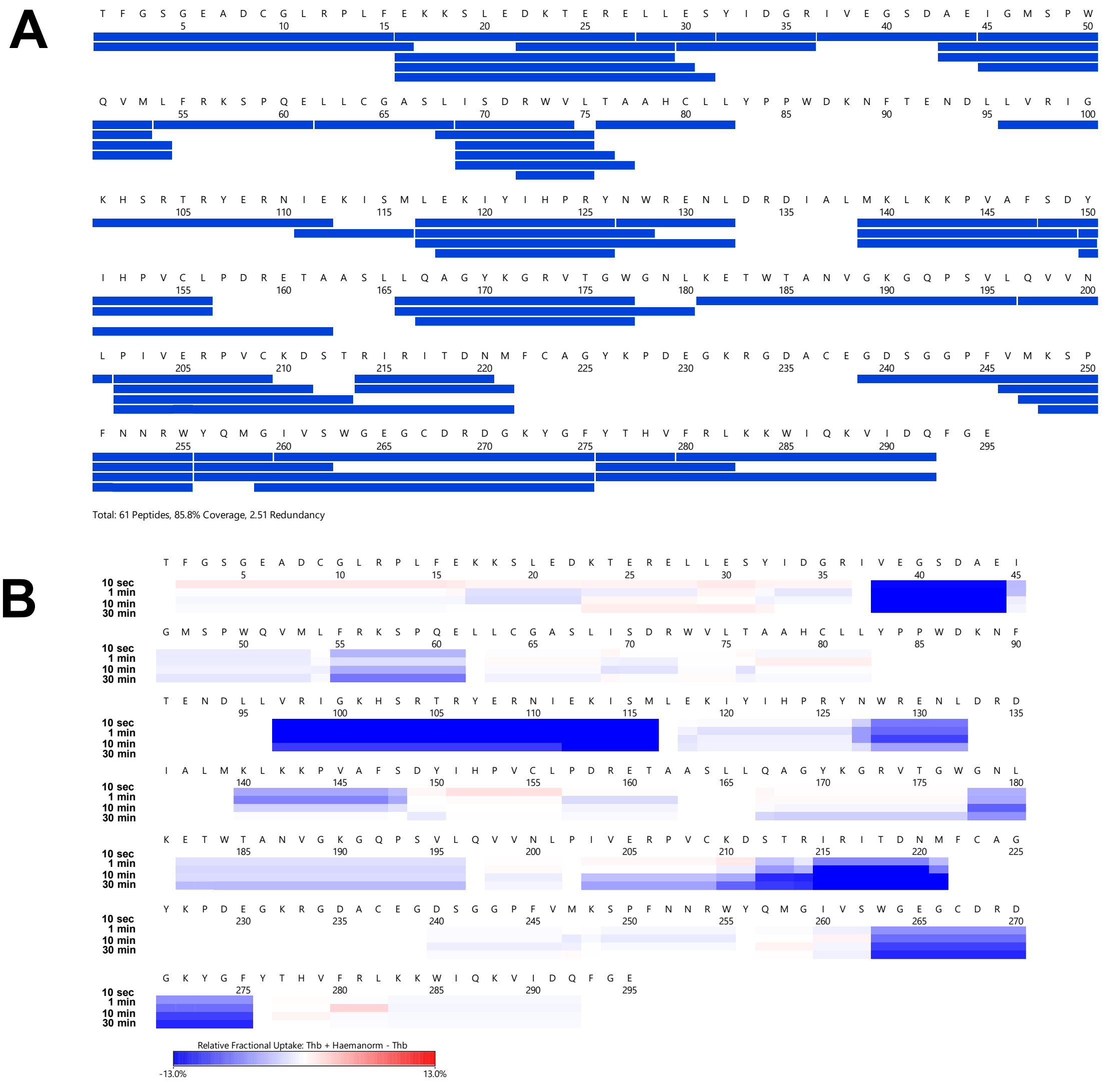

Figure S10. **HDX-MS analysis of Haemanorm binding to αT.** (**A**) Sequence coverage of αT after peptide mass fingerprint analysis with pepsin. Blue bars represent the peptic fragments that were identified, according to the analytical criteria reported in the Methods. (**B**) Difference bi-dimensional heatmap (%ΔD) of the per cent deuterium uptake (%D) of αT with and without Haemanorm, at increasing incubation times (15 sec – 30 min). %D data of αT alone were subtracted from the corresponding data obtained in the presence of saturating inhibitor concentration. The color key in the heatmap indicates %ΔD, from dark blue (at least -10% deuterium uptake) to light red, where negative %D differences indicate that those region in αT become more protected from H/D exchange in the presence of the ligand peptide, compared to αT alone. Only those regions displaying %ΔD > ± 4% were considered significant and used for building the three-dimensional heatmap reported in **Figure 9** of the main text. The heatmap was generated using 61 single and overlapping fragments. αT numbering used by the Dynamix software in HDX analysis is different from the classical chymotrypsin numbering (used for building the three-dimensional heatmap in **Figure 9**) and goes from Thr1 of the A-chain to Glu295 of the B-chain.

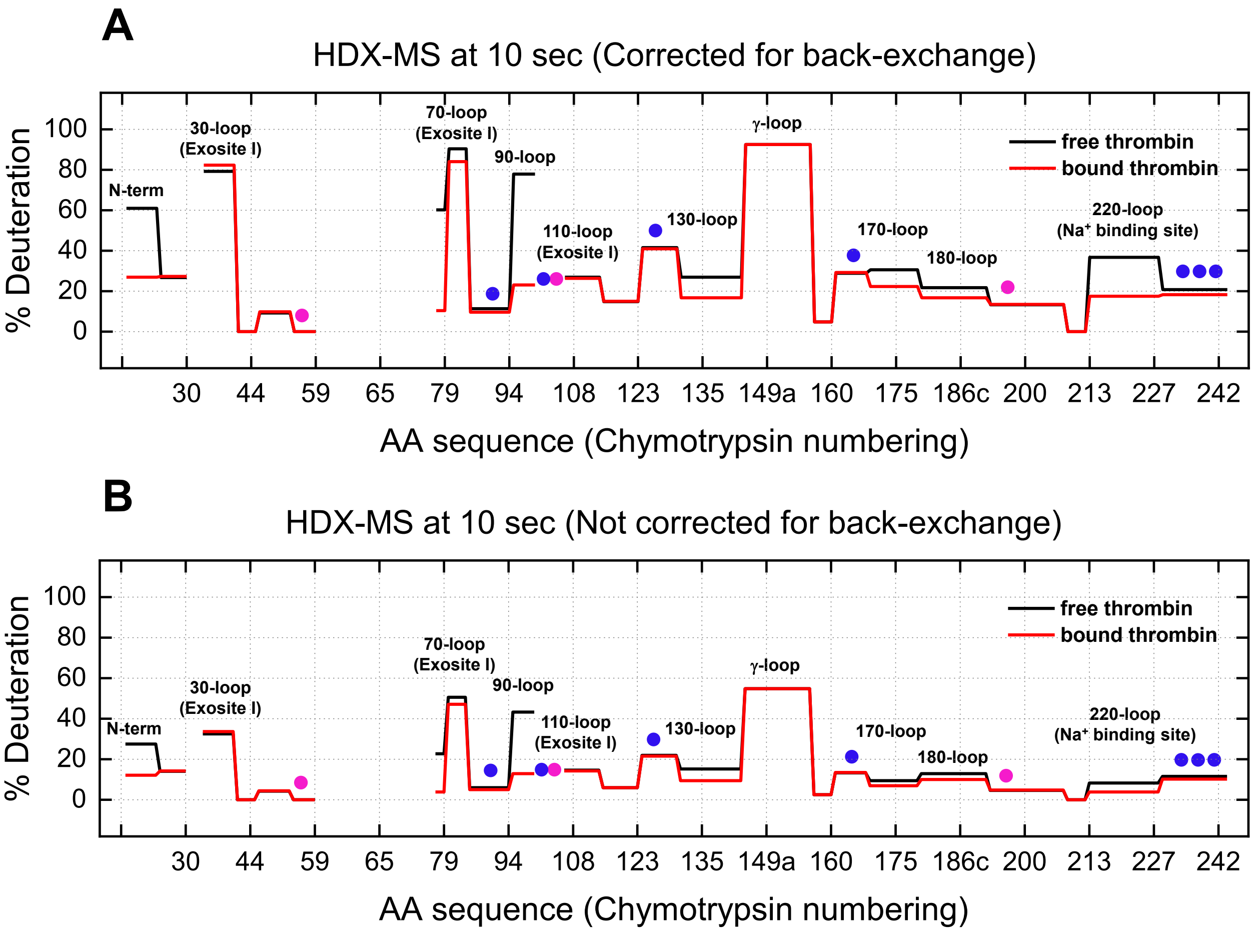

Figure S11. HDX-MS analysis of αT in the haemadin-bound (—) and unbound (—) form after 10-s H/D exchange and with (A) and without (B) correction for back-exchange. Figures show an HDX The percentage of deuterium uptake is reported as "skyline plots" (i.e. relative deuteration level *vs.* residue number) for selected thrombin peptides, represented as lines (red, for thrombin bound to haemadin, black, for free thrombin). Active site residues are indicated with magenta dots (●) while arginine residues that are comprised in exosite II are indicated with blue dots (●).

Correction for back exchange was applied according to the equation: $\%D = [(m_t - m_0) / (m_{maxD} - m_0)] \times 100$, where m_t is the observed peptide centroid mass at a given labeling time point t, m_0 is the undeuterated peptide centroid mass, and m_{maxD} is the maximally deuterated peptide centroid mass [Masson et al., 2019]. After applying back-exchange correction, the regions of αT that undergo significant changes in %D uptake remain constant, in keeping with the Δm values reported in the main text. As expected, the differences in %D uptake between the unbound and bound form of αT are enhanced, thus fully confirming the correctness of data interpretation.
